# Totally Caged Type I Pro‐Photosensitizer for Oxygen‐Independent Synergistic Phototherapy of Hypoxic Tumors

**DOI:** 10.1002/advs.202400462

**Published:** 2024-06-17

**Authors:** Qin Zeng, Xipeng Li, Jiajun Li, Mengting Shi, Yufen Yao, Lei Guo, Na Zhi, Tao Zhang

**Affiliations:** ^1^ MOE Key Laboratory of Laser Life Science & Institute of Laser Life Science Guangdong Provincial Key Laboratory of Laser Life Science College of Biophotonics South China Normal University Guangzhou 510631 China; ^2^ The Seventh Affiliated Hospital Southern Medical University Foshan Guangdong 528244 China; ^3^ School of Pharmaceutical Sciences Sun Yat‐sen University Guangzhou 510006 China; ^4^ Guangzhou Key Laboratory of Spectral Analysis and Functional Probes College of Biophotonics South China Normal University Guangzhou 510631 China

**Keywords:** background‐free imaging, hypoxia‐activatable, oxygen‐independent phototherapeutic effect, single‐pulse laser irradiation, type I pro‐photosensitizers

## Abstract

Activatable type I photosensitizers are an effective way to overcome the insufficiency and imprecision of photodynamic therapy in the treatment of hypoxic tumors, however, the incompletely inhibited photoactivity of pro‐photosensitizer and the limited oxidative phototoxicity of post‐photosensitizer are major limitations. It is still a great challenge to address these issues using a single and facile design. Herein, a series of totally caged type I pro‐photosensitizers (Pro‐I‐PSs) are rationally developed that are only activated in tumor hypoxic environment and combine two oxygen‐independent therapeutic mechanisms under single‐pulse laser irradiation to enhance the phototherapeutic efficacy. Specifically, five benzophenothiazine‐based dyes modified with different nitroaromatic groups, BPN 1−5, are designed and explored as latent hypoxia‐activatable Pro‐I‐PSs. By comparing their optical responses to nitroreductase (NTR), it is identified that the 2‐methoxy‐4‐nitrophenyl decorated dye (BPN 2) is the optimal Pro‐I‐PSs, which can achieve NTR‐activated background‐free fluorescence/photoacoustic dual‐modality tumor imaging. Furthermore, upon activation, BPN 2 can simultaneously produce an oxygen‐independent photoacoustic cavitation effect and a photodynamic type I process at single‐pulse laser irradiation. Detailed studies in vitro and in vivo indicated that BPN 2 can effectively induce cancer cell apoptosis through synergistic effects. This study provides promising potential for overcoming the pitfalls of hypoxic‐tumor photodynamic therapy.

## Graphical abstract

Enhancement of the accuracy and efficiency of activatable type I pro‐photosensitizers (Pro‐I‐PSs) in a simple and effective way remains greatly challenging. Given this, the Pro‐I‐PS BPN 2 is developed with the following features: 1) Simple and well‐defined molecular structure; 2) NTR‐triggered dramatic transition of photoactivity from completely inhibited state to highly activated state; 3) high‐contrast fluorescence/photoacoustic imaging‐guided oxygen‐independent synergistic phototherapy.

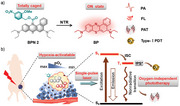

## 1 Introduction

Photodynamic therapy (PDT) has been a widely applied prospect in clinical practice owing to its advantages of minimal invasiveness and low systemic toxicity.^[^ ^1^ ^]^ The current photosensitizers (PSs) used in PDT processes are mostly based on the type II photochemical reaction, which is highly dependent on molecular oxygen (O_2_) to generate singlet oxygen (^1^O_2_) for inducing tumor cell apoptosis or necrosis.^[^ ^2^ ^]^ However, severe hypoxia is a universal feature in the solid tumor microenvironment, which ultimately leads to a suboptimal therapeutic effect.^[^ ^3^ ^]^ Distinct from O_2_‐dependent type II PDT, superoxide anion (O_2_^−•^)‐dependent type I PDT (I‐PDT) can reduce O_2_ requirement by avoiding direct and rapid O_2_ consumption, showing great potential in overcoming hypoxic cancer.^[^ ^4^ ^]^ To date, various type I PSs (I‐PSs) based on purely organic small molecules with well‐defined structures, good biocompatibility, and reproducibility have been developed for imaging‐guided I‐PDT. In spite of the progress, however, non‐selective I‐PSs might cause treatment‐related toxic side effects to adjacent normal tissue.^[^ ^5^ ^]^ Furthermore, “always‐on” I‐PSs for imaging may also induce phototoxicity during the delivery progress.^[^ ^6^ ^]^ Therefore, the development of tumor‐specific activated type I pro‐photosensitizers (Pro‐I‐PSs) with high efficacy is urgently needed and extremely challenging.

Pro‐I‐PSs can discriminate healthy cells from the diseased by turning off their photosensitizing activity in normal tissue and turning it on in tumors, thereby reducing nonspecific damage to neighboring normal cells.^[^ ^7^ ^]^ Recently, a few Pro‐I‐PSs with type I photosensitivity activated by specific stimuli have been developed to improve tumor targeting.^[^ ^8^ ^]^ However, the photoactivities of most Pro‐I‐PSs that are not minimized before activation can still cause nonspecific damage. Therefore, a Pro‐I‐PSs activation strategy with the biomarker‐specifically triggered dramatic switch in its photosensitivity from a completely inhibited to a highly reactive state offers important promise in the field of precision medicine.^[^ ^9^ ^]^ Briefly, totally caged Pro‐I‐PSs for improving the precision of I‐PDT are highly desirable, especially near‐infrared light excited.

Objective to the efficiency of tumor treatment, the antitumor effects of a single I‐PDT are unsatisfactory because cancer cells can adapt to oxidative stress by activating antioxidant systems and various anti‐apoptosis signaling pathways.^[^ ^10^ ^]^ Combining I‐PDT with other treatments can effectively improve the overall therapeutic effect by harboring the collective merits of respective individual treatments.^[^ ^11^ ^]^ However, most combination therapeutic strategies are based on complex materials with multiple components encapsulated in or conjugated to carriers, generally resulting in unknown toxicity and irreproducibility.^[^ ^12^ ^]^ Thus, there is an urgent need to develop a single active component that can provide different therapeutic mechanisms for cancer combination therapy. Previous studies by our group have shown that photosensitizers can combine photophysical and photochemical damage under pulsed light irradiation to simultaneously generate acoustic cavitation and reactive oxygen species (ROS), thereby significantly improving the phototherapy effect of hypoxic tumors.^[^ ^13^ ^]^ The photoacoustic cavitation effect is that under pulsed light irradiation, the photosensitizer absorbs photon energy to trigger the generation, growth, and subsequent collapse of cavitation bubbles, thereby causing a relatively strong shock wave and causing damage to the lesion area by inducing this instantaneous mechanical force.^[^ ^14^ ^]^ Given the above, the development of totally caged Pro‐I‐PSs with multiple functions of targeting, diagnosis, and collaborative treatment would be attractive candidates for future cancer treatments.

**Scheme 1:**
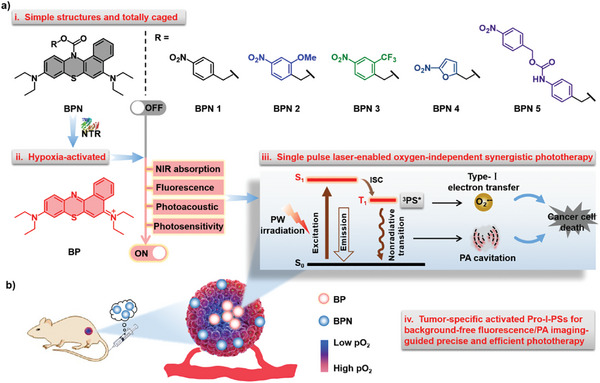
Schematic illustration of hypoxia‐responsive Pro‐I‐PSs BPN to specifically eradicate hypoxic tumors by a single‐pulse laser‐enabled O_2_‐independent phototherapeutic effect.

Herein, we rationally designed and constructed a series of novel hypoxia‐responsive Pro‐I‐PSs (BPN), in which nitroaryl group can be reduced by nitroreductase (NTR) to release free I‐PS (benzophenothiazine, BP) for oxygen‐independent synergistic phototherapy in hypoxic tumors (**Scheme** **1**). Hypoxia can cause an increase in cellular reductive stress, resulting in an excess expression of NTR, azoreductase, quinone reductase, etc.^[^ ^15^ ^]^ Therefore, NTR is considered a tumor‐associated stimulus for the activation of drug release, and its level is directly related to the degree of hypoxia. Specifically, BPN 2 has been established with the following features: 1) well‐defined composition and molecular structure; 2) its photoactivity can undergo a dramatic transition from completely inhibited state to highly activated upon triggered by NTR; 3) tumor‐specific background‐free fluorescence/photoacoustic (PA) imaging; 4) ability to produce oxygen‐independent I‐PDT effect and PA cavitation in the target tumor under single‐pulse laser irradiation. These characteristics could make BPN 2 promising to enhance the effectiveness and precision of phototherapy in hypoxic tumors.

## 2 Results and Discussion

### 2.1 Design and Synthesis of BPN 1–5

**Figure 1:**
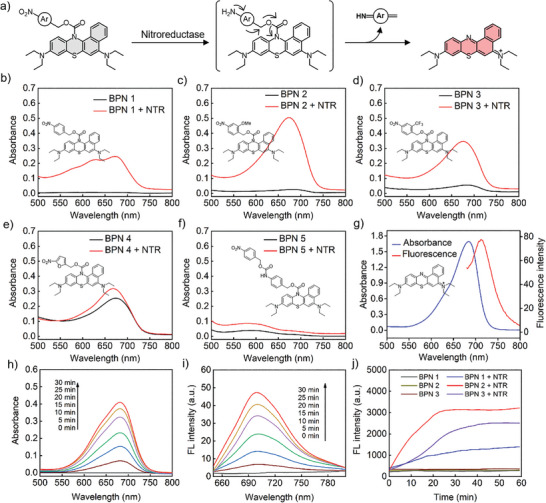
a) Principle of hypoxia‐responsive Pro‐I‐PSs activated with NTR. b–f) Absorbance spectra of BPN 1−5 (10 µm) before and after reaction with NTR (10 µg mL^−1^). g) Absorbance and fluorescence spectra of I‐PS BP. Kinetic UV–vis absorption h) and fluorescence i) spectral of BPN 2 (10 µm) reacted with NTR (10 µg mL^−1^). j) Time‐dependent fluorescence emission intensity (Ex = 680 nm, Em = 720 nm) of BPN 1−3 reacted with NTR.

Appropriate Pro‐I‐PS for the treatment of hypoxic solid tumors should satisfy the prerequisites of the high selectivity and oxygen‐independent phototherapeutic effect. Previously, our group designed a series of methylene blue‐based activatable photosensitizers with completely inhibited photosensitivity, which achieved efficient and precise type II PDT of tumors without major side effects.^[^ ^16^ ^]^ Recently, other groups demonstrated that BP, an analog of methylene blue, can undergo a type I photochemical mechanism to generate O_2_^−•^, enhancing hypoxic tumor therapy in a low O_2_‐dependent manner.^[^ ^17^ ^]^ Given that, in the present study, the nitroaryl group as an NTR switch was coupled to the nitrogen of the BP ring via a carbamate linker to obtain Pro‐I‐PSs. The photoactivity of Pro‐I‐PSs is completely inhibited due to the destruction of the conjugated structure of BP but could be restored by the reducing action of NTR (**Figure** **1**a). Enlightened by the previous studies on hypoxia‐activated nitroaryl prodrugs,^[^ ^18^ ^]^ we designed the different nitroaryl groups and evaluated their reactivity toward NTR to determine the optimal Pro‐I‐PS structure. Specifically, we synthesized five benzothiaphenazine‐based NTR‐activatable Pro‐I‐PSs BPN 1–5 including several nitroaryl substrates, such as 4‐nitrophenyl, 2‐methoxy‐4‐nitrophenyl, 2‐trifluoromethy‐4‐nitrophenyl, 5‐nitrofuranyl and 4‐[[[(4‐nitrobenzyl)oxy]carbonyl]amino]benzyl. The synthesis and characterization of BPN 1–5 are detailed in the supporting information (Figures S1–S18, Supporting Information).

With these NTR‐activatable Pro‐I‐PSs in hand, we first evaluated the absorption and fluorescence responses of these five Pro‐I‐PSs to NTR under physiological conditions in the presence of nicotinamide adenine dinucleotide (NADH). NADH as an electron donor can enable NTR to catalyze the reduction of nitro compounds to the corresponding amine compounds under hypoxic conditions.^[^ ^19^ ^]^ As shown in Figures 1b–f and S19 (Supporting Information), BPN 1–3 showed rather low absorption and emission peaks in the 500–800 nm wavelength range before reacting with NTR. While adding NTR to these probe solutions, they exhibited obviously strong absorption peaks at 680 nm and fluorescence peaks at 705 nm, and the reaction solution also changed from colorless to cyan. Among them, the 2‐methoxy‐4‐nitrophenyl‐based Pro‐I‐PS BPN 2 showed the strongest response in absorbance and fluorescence intensity, which was much higher than BPN 1 and BPN 3. In contrast, BPN 4 and BPN 5 showed only negligible absorbance and fluorescence responses in the presence of NTR. Notably, the absorption and fluorescence spectra of BPN 2 after reacting with NTR were consistent with the I‐PS BP, indicating that the reaction of BPN 2 with NTR can be more complete than other Pro‐I‐PSs (Figure 1g). Moreover, we also further investigated the kinetics of the interaction between BPN 1–3 and NTR by UV–vis and fluorescence spectroscopy. As shown in Figure 1h,i, the absorption and emission peaks of BPN 2 increased significantly with time after adding NTR. More importantly, the fluorescence kinetics experimental results showed that the fluorescence of BPN 2 increased the fastest after adding NTR compared with BPN 1 and BPN 3 (Figure 1j). Taken together, these studies demonstrate that 2‐methoxy‐4‐nitrophenyl modified BP (BPN 2) is more effective than 4‐nitrophenyl, 2‐trifluoromethy‐4‐nitrophenyl, 5‐nitrofuranyl and 4‐({[(4‐nitrobenzyl)oxy]carbonyl}amino)benzyl in NTR‐catalyzed reduction reactions. More importantly, these results suggest that 2‐methoxy‐4‐nitrophenyl is superior to 4‐nitrophenyl, which has been the frequently used group of nitroaryl in the literature,^[^ ^20^ ^]^ thus providing new guidance for the development of more effective NTR‐activatable Pro‐I‐PSs.

### 2.2 Theoretical Studies on the Binding Pattern of Pro‐PSs with NTR

**Figure 2:**
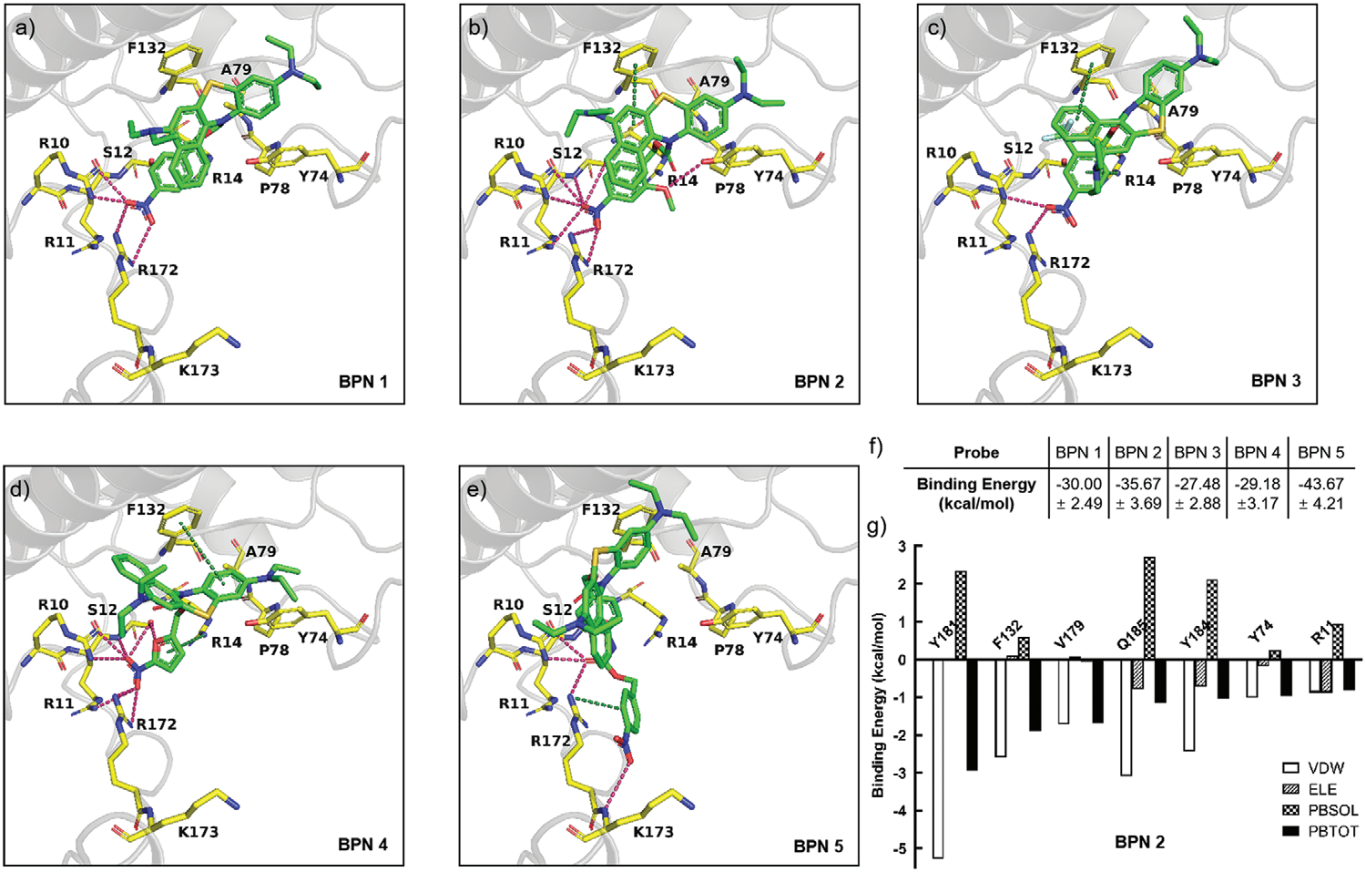
Theoretical studies on the binding pattern of Pro‐I‐PSs with NTR. Molecular docking models of a) BPN 1, b) BPN 2, c) BPN 3, d) BPN 4, and e) BPN 5 with NTR. The backbones of BPN 1–5 are shown in green, and the side chains of NTR are shown in yellow. The cartoon structures of NTR are shown in gray. N, O, and S atoms in the docking models are shown in blue, red, and yellow, respectively. The amino acid residues of NTR are labeled. Hydrogen bonds are indicated with purple dotted lines. f) Calculated binding free energies are estimated by the MM/PBSA method. g) Decomposition of the key residues contributions to the binding free energy for the complex of 4DN2 with probe BPN 2. VDW: the Van der Waals free energy, ELE: the electrostatic free energy, PBSOL: the solvation free energy calculated in the PB method, PBTOT: the total free energy calculated in the PB method.

Molecular docking and molecular dynamics (MD) simulations were performed to get a deep understanding of the relationship between the Pro‐I‐PSs structure and NTR detection ability.^[^ ^21^ ^]^ The efficient substrate binding as the first step is critical for the NTR‐catalyzed reduction before the following catalytic reduction, and product departure.^[^ ^22^ ^]^ As shown in **Figure** **2**a–e, the large BP skeleton of all Pro‐I‐PSs is situated in the hydrophobic interspace of NTR surrounded by amino acid residues Tyr74, Pro71, Ala79, Val131, and Phe132, which interacts with Pro‐I‐PSs via hydrophobic interactions and the π–π interactions.^[^ ^20^ ^]^ More importantly, multiple hydrogen bonds are involved between the nitro groups substituted on the side chain of BPN 1–4 and the amino acid residues of NTR, containing Arg10, Arg11, Ser12, and Arg172, which are critical to form a transition state for the catalytic reduction process.^[^ ^18b^ ^]^ In contrast, the absolute values of predicted binding free energies (MM‐PBSA total energy) of different Pro‐I‐PSs account for the difference in binding affinity (Figure 2f), indicating that the key reactive nitro groups on different substituted side chains revealed different responsive effects. Indeed, BPN 1, BPN 2, BPN 3, and BPN 4 containing 4‐nitrophenyl, 2‐methoxy‐4‐nitrophenyl, 2‐trifluoromethyl‐4‐nitrophenyl, and 5‐nitrofuranyl, respectively, showed the binding free energies among −35.67 to −27.48 kcal mol^−1^. Thereinto, BPN 2 with‐OMe at the ortho position showed the lower MM/PBSA binding free energy, consisting of its better catalytic response to NTR, which was possibly attributed to the strong interactions with amino acid residues including Arg11, Tyr74, and Phe132 (Figure 2g). Furthermore, the structural feature of BPN 2 with the ─OMe group afforded additional hydrogen binding with Tyr74 of NTR compared with that of BPN 1 as shown in Figure 2b. The residue decomposition results of BPN 2 toward NTR disclosed a potential high binding affinity through interactions between the nitro group and residue Arg11, a hydrogen bond interaction between ─OMe group and Tyr74 as well as a π–π interaction between the BP skeleton and Phe132, highlighting the more favorable catalytic reaction response of BPN 2 toward NTR. Although BPN 5 revealed the MM/PBSA binding free energy as low as −43.67 kcal mol^−1^ indicating higher binding affinity toward NTR, the nitro group on the overlong side chain hardly inserted into the catalytic reaction site of NTR to match multiple hydrogen bonds with Arg10, Arg11, Ser12, and Arg172 (Figure 2e), resulting in insensitivity to NTR.

### 2.3 Optical Characteristic of BPN 2 to NTR

**Figure 3:**
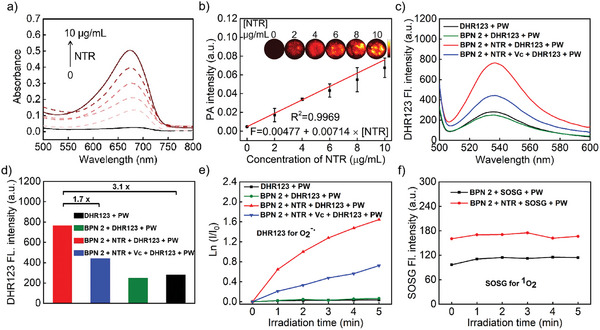
a) Absorbance spectra of BPN 2 (10 µm) toward varied concentrations of NTR. b) PA images and PA intensities of BPN 2 (10 µm) toward varied concentrations of NTR. c) Fluorescence spectra of DHR123 for O_2_^−•^ detection. d) The fluorescence intensity of DHR123 after different treatments. e) Comparison of O_2_^−•^ generation as a function of irradiation time. f) The fluorescence intensity of singlet oxygen sensor green (SOSG) for ^1^O_2_ detection.

Considering BPN 2 has been demonstrated as the optimal Pro‐I‐PSs for NTR activation, the absorption and fluorescence responsiveness of BPN 2 toward NTR was further investigated by UV‐vis and fluorescence spectroscopy. The result exhibits that the absorption and emission peaks of BPN 2 increased significantly with the increase of NTR concentration (**Figure** **3**a; Figure S20, Supporting Information). The reaction selectivity of BPN 2 was further studied by evaluating various latent interfering species. As shown in Figure S21 (Supporting Information), compared with the blank group, only the NTR group in the presence of 500 µm NADH showed obvious fluorescence enhancement. In contrast, other interfering species including Cys, GSH, NaHS, H_2_O_2_, Vc, ClO^−^, NaCl, KCl, CaCl_2_, glucose, and Na_2_S_2_O_3_ did not cause significant fluorescence turn‐on response, thus confirming the specificity of BPN 2 toward NTR.

After demonstrating the specific absorption and fluorescence response of BPN 2 toward NTR, the PA property of BPN 2 was further investigated. As shown in Figure 3b, the PA signals of the BPN 2 solution at 680 nm with different concentrations of NTR exhibits that the PA intensity of the BPN 2 solution increased gradually under the pulse‐wave (PW) irradiation as the NTR concentration was increased from 0 to 10 µg mL^−1^. Then, the photosensitizing capability of BPN 2 was investigated by measuring the photogenerated ROS under different conditions. On the one hand, dihydrorhodamine123 (DHR123) was employed as an O_2_^−•^ probe to detect O_2_^−•^ production. DHR123 is non‐fluorescent but can respond to O_2_^−•^ emitting strong green fluorescence. As shown in Figure 3c, the group of BPN 2 and control (containing DHR123) have negligible fluorescence change upon 680 nm PW irradiation, suggesting that BPN 2 did not generate O_2_^−•^. It is worth noting that under the same irradiation conditions, the fluorescence intensity of DHR123 increased by 3.1 times when BPN 2 reacted with NTR, indicating efficient O_2_^−•^ generation. In addition, as expected, the fluorescence intensity of the group of BPN 2 + NTR treated with Vc (vitamin C, a radical scavenger) was reduced by 1.7‐fold, further demonstrating that O_2_^−•^ generation can indeed enhance the signal of DHR123 (Figure 3d). Similarly, after the reaction of BPN 2 with NTR, the fluorescence intensity of DHR123 gradually increased with the irradiation time from 0 to 5 min, further confirming the above results (Figure 3e).

On the other hand, we also further investigated the generation of ^1^O_2_ and OH• with SOSG (singlet oxygen sensor green, the specific indicators for ^1^O_2_) and BA (benzoic acid, the specific indicators for OH•),^[^ ^23^ ^]^ respectively. As shown in Figures 3f and S22a,b (Supporting Information), under PW irradiation, BPN 2 has no apparent fluorescence change with or without NTR, indicating that no other ROS were produced. To further verify that BA can detect the generation of OH•, we have added the positive control group: H_2_O_2_ (50 µm) + different concentrations of Fe^2+^ (0–10 µm). In this reaction, ferric ions are oxidized to ferrous ions, and H_2_O_2_ decomposes to produce OH•. Then, we investigated the generation of OH• using BA. As shown in Figure S22c (Supporting Information), the group of H_2_O_2_ + Fe^2+^ showed a significant increase in fluorescence with the Fe^2+^ concentration increase, indicating the generation of OH•. These results demonstrated that BA can be used to detect the production of OH•. In addition, electron spin resonance (ESR) spectroscopy was employed to confirm the generation of OH•. In this method, 5, 5 dimethyl‐1‐pyrroline‐N‐oxide (DMPO) were applied as spin‐trap agents for OH•. As shown in Figure S22d (Supporting Information), under light irradiation, BPN 2 has no apparent characteristic paramagnetic adduct with or without NTR, indicating that no OH• were produced. Therefore, these results reveal that BPN 2 activated by NTR can undergo the I‐PDT mechanism (electron transfer photochemical process) under PW irradiation.

### 2.4 Intracellular Activation and Phototoxicity of BPN 2

**Figure 4:**
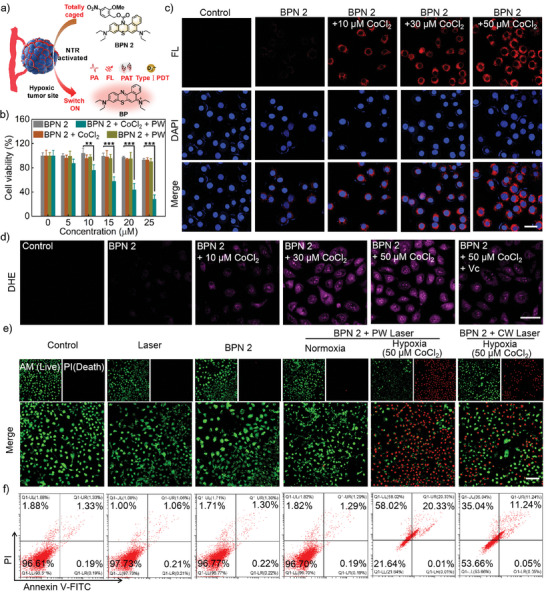
a) Schematic illustration of the BPN 2 for NTR‐triggered oxygen‐independent phototherapeutic effect against hypoxic tumor. b) The cell viability of EMT6 cells incubation with diverse concentrations of BPN 2 after different treatments. c) Fluorescence imaging of endogenous NTR activities in EMT6 cells by using BPN 2. Scale bar = 50 µm. d) Fluorescence imaging of cellular O_2_^−•^ after exposure to 0.5 W cm^−2^ PW light dose in different treatments. Scale bar = 50 µm. e) Confocal fluorescence imaging of live/dead cell staining after different treatments. Scale bar = 100 µm. f) Flow cytometric analysis of cells after different treatments.

We next investigate the suitability of BPN 2 as a hypoxia‐activatable Pro‐I‐PS in biological systems (**Figure** **4**a). The internalization and responsiveness of BPN 2 were first assessed in NTR‐overexpressed cancer cells. Because the cobalt chloride (CoCl_2_) was evidenced to elevate the level of NTR in living cells,^[^ ^24^ ^]^ EMT6 mouse mammary carcinoma cells were incubated with CoCl_2_ to mimic the hypoxia microenvironments of the tumor. we first explored the effect of CoCl_2_ on cell viability to determine the appropriate CoCl_2_ concentration for cells, and the results showed that no significant cytotoxicity was caused by CoCl_2_ (Figure S23, Supporting Information). 3‐(4,5‐dimethylthiazol‐2‐yl)‐2,5‐diphenyltetrazolium (MTT) assay was carried out to further investigate the cytotoxicity of BPN 2. As shown in Figure 4b, without photoirradiation, the BPN 2 treated group exhibit negligible cytotoxicity in both normoxia (without CoCl_2_) and hypoxia (with CoCl_2_). Under 680‐nm PW irradiation, no obvious inhibitory effect was observed when BPN 2 was coculture with EMT6 cells without CoCl_2_ treatment. By contrast, in the presence of CoCl_2_, BPN showed significant concentration‐dependent cytotoxicity, and the cell viability was decreased to 28.3% at a dose of 25 µm when exposure to the laser, indicating that BPN 2 can specifically respond to NTR and efficiently kill hypoxia tumor cells under laser irradiation. The capacity of BPN 2 to image intercellular NTR was then examined. As shown in Figure 4c, cells incubated with BPN 2 showed ignorable red fluorescence, while cells induced by CoCl_2_ revealed significant red emission after incubation with BPN 2. In addition, the fluorescent signal gradually increased with the extension of CoCl_2_ concentration, indicating that BPN 2 was successfully internalized into cancer cells and activated by NTR. To evaluate the I‐PDT efficacy, intracellular O_2_^−•^ production was measured using dihydroethidium (DHE). As shown in Figure 4d, the control group revealed no fluorescence, while the fluorescence intensity of BPN 2‐treated EMT6 cells under light irradiation was enhanced with the increase of the added CoCl_2_ concentration. In addition, the fluorescence intensity of the group of BPN 2 + 50 µm CoCl_2_ treated with 500 µm Vc was reduced significantly, further demonstrating that BPN 2 can achieve NTR‐controllable O_2_^−•^ generation in hypoxia cancer cells.

Next, phototherapy experiments were further performed on EMT6 cells in normoxia and hypoxia to explore the hypoxia‐triggered oxygen‐independent phototherapeutic effect of BPN 2. Cells were co‐stained with calcein‐AM (live cell, green fluorescence) and PI (dead cell, red fluorescence) and imaged by confocal microscopy. As shown in Figure 4e, cells treated with PW Laser and BPN 2 respectively remained survival compared to the control group. No significant toxicity was found in the BPN 2 + PW Laser group under normoxic conditions (without CoCl_2_). Interestingly, the BPN 2 + PW Laser group exhibited remarkable cell death under hypoxic conditions (with CoCl_2_), which was superior to the BPN 2 + CW (continuous‐wave) Laser group. Among them, BPN 2 demonstrated the I‐PDT effect alone after specifically responding to NTR when exposed to a 680‐nm CW laser. The efficacy of phototherapy was also quantified by flow cytometric analysis, using Annexin V‐FITC/propidium iodide (PI) as a marker to differentiate apoptotic and necrotic cells. It was found that ≈20.34% of cells were apoptotic and ≈58.02% of cells were necrotic in the BPN 2 + PW Laser group under hypoxic conditions (with CoCl_2_), implying that the cell damage was caused by both necrosis and apoptosis (Figure 4f). These results indicated that hypoxia‐triggered BPN 2 can integrate dual photomechanical and photochemical damage effects under PW irradiation, resulting in a higher therapeutic efficiency toward hypoxic tumor cells over the I‐PDT effect alone.

To verify the photoacoustic cavitation capability of BPN 2, intracellular generation of photocavitation was then investigated by monitoring the morphology of hypoxia‐treated EMT6 cells incubated with BPN 2 under the PW and CW laser irradiation. 2, 7‐dichloro fluorescein diacetate (DCFH‐DA) was used to detect the production of ROS. As shown in Figure S24 (Supporting Information), compared with the control group, BPN 2 showed obvious DCFH fluorescence under PW or CW laser irradiation, proving that BPN 2 has a significant photodynamic effect. More importantly, we directly observed that the cell morphology of cells treated with BPN 2 obviously changed from a plump state to a fragmented state after irradiation with PW (680 nm, 0.5 W cm^−2^), indicating the high efficiency of BPN 2‐induced photoacoustic cavitation effect. In addition, no fluorescence was observed in cells treated with BPN 2 and Vc under PW irradiation, but its cell morphology showed slight damage. Thus, BPN 2 exhibited a remarkable combination of photodynamic and photoacoustic cavitation effects.

To further evaluate the ability of BPN 2 for oxygen‐independent synergistic I‐PDT and PA cavitation therapy (PAT) of hypoxic tumors, we compared the efficacy of the BPN 2 + PW Laser (I‐PDT + PAT) with BPN 2 + CW Laser (I‐PDT) and BPN 2 + Vc + PW Laser (excluding I‐PDT and preserving PAT). MTT was also performed to quantitatively assess BPN 2‐induced proliferation inhibition in hypoxic EMT6 cells. As shown in Figure S25 (Supporting Information), the cell viability of the BPN 2‐treated group was found to be dramatically decreased to 28.0% under PW laser irradiation, which was superior to either I‐PDT (down to 49.1%) or PAT (down to 40.0%) alone. These results suggest that BPN 2 can efficiently kill hypoxic tumor cells via the synergistic effect of I‐PDT and PA cavitation.

### 2.5 In Vivo Imaging and Tumor Inhibition of BPN 2

**Figure 5:**
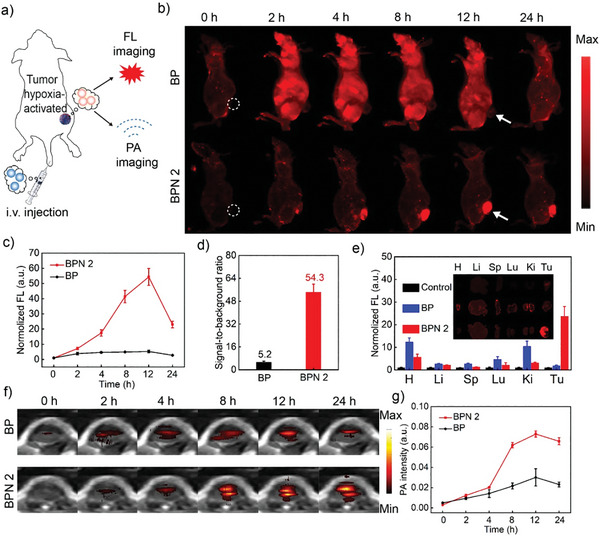
a) Experimental design of NTR imaging in vivo. b) In vivo fluorescence imaging after intravenous injection of BPN 2 or BP. c) Corresponding fluorescence intensity of tumors as a function of post‐injection time of BPN 2 or BP. d) The signal‐to‐background ratio at 12 h post intravenous injection of BPN 2 or BP. e) Ex vivo fluorescence imaging of dissected tumors and organs from mice in different groups at 24 h post‐injection and quantified fluorescence intensities. f) In vivo PA after intravenous injection of BPN 2 or BP. g) Corresponding PA intensity of tumors as a function of post‐injection time of BPN 2 or BP.

The in vivo tumor fluorescence and PA imaging ability of hypoxia‐activatable BPN 2 were validated in subcutaneous EMT6 xenograft mice models. Mice were injected intravenously (i. v.) with BPN 2 and BP for in vivo fluorescence and PA imaging (**Figure** **5**a). Before using BP and BPN 2 for animal experiments, the biocompatibility of BP and BPN 2 was investigated by serum biochemical and hematological. It was suggested that BP and BPN 2 did not make abnormal changes during blood circulation (Figure S26, Supporting Information). Then, the fluorescence imaging of those subcutaneous EMT6 xenograft mice was recorded at different times. As shown in Figure 5b,c, after the injection of BPN 2, the fluorescence signal at the tumor site gradually increased. However, with BP, the fluorescence signal travels quickly throughout the body. Due to the rapid clearance of BP in the body, the fluorescence of the cancer area faded rapidly throughout the observation process. Importantly, compared with BP, the tumor was specifically “illuminated” 12 h after i. v. administration of BPN 2, which allowed it to be clearly distinguished from surrounding normal tissues. At this point, the signal‐to‐background ratio (SBR) was as high as 54.3, significantly higher than BP (SBR: 5.2) (Figure 5d). Ex vivo fluorescence imaging of excised tissues from different groups further confirmed that the mice treated with BPN 2 had obvious fluorescence signals at tumor sites, while other organs (heart, liver, spleen, lung, and kidney) showed very weak fluorescence. For BP‐treated mice, the tumor tissue was similar to that of the control group, showing negligible fluorescence signals (Figure 5e).

Given that hypoxia‐activatable BPN 2 has large absorption changes in the NIR region, its high‐contrast PA imaging in the tumor tissue can provide complementary information for fluorescence imaging. As shown in Figure 5f,g, the PA signal in the tumor area was significantly increased after tail vein injection of BPN 2 and reached its maximum value within 12 h with 11.7‐fold normalized intensity. This behavior is in good agreement with the fluorescence imaging results. In addition, the lower imaging contrast and rapid clearance in the tumor site were also observed for the BP. To further illustrate the behavior of the BPN 2 in vivo, a blood circulation investigation was performed (Figure S27, Supporting Information). The results showed that the blood levels of the BPN 2 reduced gradually over time with the plasma half‐life determined as 8.2 ± 0.7 h, but it was maintained at a relatively effective concentration even at 24 h postinjection. Considering these experimental results, BPN 2 undoubtedly serves as a specific hypoxia‐activated fluorescence/PA imaging agent for guiding the synergistic anticancer effect of I‐PDT and PA cavitation in vivo.

**Figure 6:**
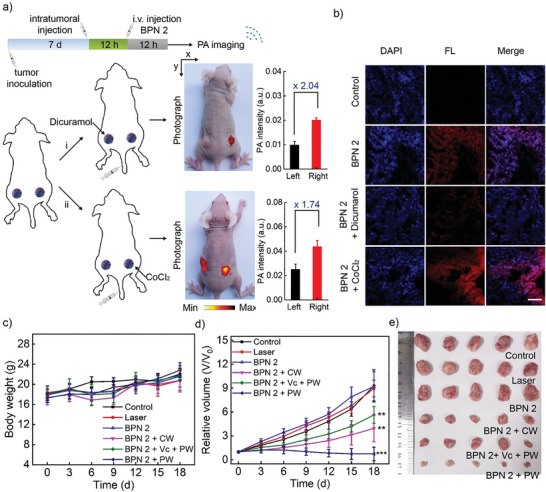
a) Photograph and PA imaging of a mouse with two EMT6 tumors on the left and right 12 h after intravenous injection of BPN 2. Above: the left tumor was injected with dicoumarin as an inhibitor of NTR; the right tumor was without treatment. Below: the left tumor was without treatment; the right tumor was injected with CoCl_2_ to elevate the level of NTR. b) BP fluorescence imaging of the tumor with different treatments. c) The body weight variation of tumor mice after different treatments. d) The tumor growth curves over 18 days after different treatments. e) Photographs of tumor tissues received different treatments for 18 days.

Moreover, to further demonstrate the high specificity and sensitivity of BPN 2 for hypoxia solid tumors in vivo, we designed a double tumor‐bearing mouse model to investigate the ability of BPN 2 for high‐contrast PA imaging. As shown in **Figure** **6**a, first when dicuramol (an NTR inhibitor) was injected intratumorally to the left side of the double‐tumor model for 12 h of pretreatment, the experimental results of PA imaging after 12 h of tail vein injection of BPN 2 clearly illustrated that the right tumor exhibited ≈2.04‐fold higher PA intensity than the left side. Second, after intratumorally administrating CoCl_2_ to the right sides of the double‐tumor model for 12 h of pretreatment, the PA intensity after 12 h of tail vein injection of BPN 2 was recorded that the right tumor exhibited ≈1.74‐fold higher than the left side. Encouragingly, the tumor hypoxia responsiveness of BPN 2 in vivo was also further confirmed by collecting the abovementioned differently treated tumor tissues. The images of tumor sections from BPN 2‐injected mice showed obviously fluorescent signals compared with controls (Figure 6b). While in the CoCl_2_ pretreatment group, the images of tumors showed higher fluorescence signals. Conversely, as expected, the images of tumors pretreated with NTR inhibitors showed a significant reduction in fluorescence, suggesting that intratumoral NTR is key to effectively restoring the fluorescence of BPN 2. Taken together, it was revealed that BPN 2 could be specifically activated by NTR overexpressed in tumors.

Finally, the in vivo BPN 2 antitumor potency was investigated in EMT6 xenograft mice models. Initially, mice were randomly divided into six groups (*n* = 5), including the control, Laser, BPN 2, BPN 2 + CW (I‐PDT), BPN 2 + Vc + PW(excluding I‐PDT and preserving PAT), and BPN 2 + PW (I‐PDT + PAT) groups. During the 18‐day treatment period, the weights of the body and volume of tumors were monitored every 3 days. As shown in Figures 6c and S28 (Supporting Information), no obvious differences or abnormalities were found in the body weight of mice in all groups. However, it was found that the tumor volumes of mice were continuously increasing in the control, laser, BPN 2, and BPN 2 + Vc + CW group (the addition of Vc inhibits the PDT effect). On the contrary, tumors in the BPN 2 + CW and BPN 2 + Vc + PW groups exhibited 57.6% and 38.3% growth inhibition, which should be derived from the effect of I‐PDT and PAT, respectively. More importantly, the BPN 2 + PW group exhibited complete tumor ablation (92.1%), which should benefit from the effect of I‐PDT synergistic PA cavitation (Figure 6d,e). Besides, when we pretreated the tumors with an NTR inhibitor (dicumarol), the overall antitumor effect was significantly reduced, accompanied by a decrease in tumor growth inhibition to 33.1%, indicating the feasibility of the proposed hypoxia‐activatable Pro‐I‐PS BPN 2 in vivo (Figure S29, Supporting Information). To further evaluate the outstanding BPN 2 antitumor potency, hematoxylin & eosin (H&E) staining of tumor sections were used to appraise the apoptosis of tumor cells after the phototreatment on day 18. As shown in Figure S30 (Supporting Information), compared with other groups, the necrosis rate of tumor cells (shrinkage/fragmentation of the nucleus and reduced nuclear density) was the highest in tumor tissues of the BPN 2 + PW group. To further demonstrate the apoptosis of tumor tissue, the terminal deoxynucleotidyl transferase dUTP nick end labeling (TUNEL) assay was used to assess. As shown in Figure S30b (Supporting Information), TUNEL assay results showed that the green fluorescence of the BPN 2 + PW Laser group was obviously enhanced, suggesting that the inhibitory effect of BPN 2 on tumor growth was caused by both necrosis and apoptosis. Moreover, no apparent histological damage was observed in the sections of major organs, indicating that BPN 2 has excellent biocompatibility and safety (Figure S31, Supporting Information). In brief, hypoxia‐activatable BPN 2 utilizes the I‐PDT and PA cavitation effects triggered by single‐pulse laser irradiation to achieve tumor‐specific enhanced synergistic therapeutic effects.

## 3 Conclusion

In conclusion, we have designed and synthesized a series of benzophenothiazine‐based dyes with different nitroaromatic modifications for the development of hypoxia‐activatable Pro‐I‐PSs. Among them, experimental and theoretical investigations revealed that the pro‐photosensitizer BPN 2 with 2‐methoxy‐4‐nitro‐modification exhibited the best response to the NTR, which was then successfully used to eradicate the hypoxic tumors. BPN 2 has completely inhibited photoactivity due to the destruction of its conjugated structure, while it can thoroughly recover all its characteristic photophysical properties in the presence of NTR, including its absorbance, fluorescence, PA, and I‐PDT effect. Moreover, BPN 2 can localize to cancer xenografts in vivo and is activated by the endogenous NTR to generate high tumor‐to‐normal imaging contrast after intravenous injection. The high levels of NTR in hypoxia tumor cells activated BPN 2 to release free I‐PS, which can produce oxygen‐independent I‐PDT and PA cavitation effects under a single‐pulsed laser irradiation. The synergistic effect of oxidation and mechanical damage afforded a powerful phototherapeutic effect to the hypoxic tumors. In short, with BPN 2, we achieved tumor‐specific imaging and efficient and precise phototherapy. Our hypoxia‐activatable type‐I pro‐photosensitizing scaffold provides a new strategy for further study in conquering hypoxic tumors.

## 4 Experimental Section

### Materials and Instrumentation

All reagents and solvents were purchased from Bidepharm and Energy chemical (China) suppliers and used directly without purification. The *Escherichia coli* nitroreductase (NTR) and nicotinamide adenine dinucleotide (NADH) were obtained from Sigma–Aldrich. Annexin V‐FITC/PI apoptosis detection kit, Intracellular O_2_^−•^ detector dihydroethidium (DHE), the superoxide assay kit (dihydrorhodamine 123, DHR123) and benzoic acid (BA) were purchased from Beyotime biotechnology. Singlet oxygen sensor green (SOSG) was purchased from Thermo Fisher Scientific Co, Ltd. (China). Cell‐counting kit‐8 (CCK‐8) was acquired from Dojindo Laboratories (Kumamoto, Japan). Calcein‐AM)/PI were purchased from Sigma–Aldrich Corporation (MO, USA).

### Characterizations

The optical characteristics of BPN 1–5 were investigated by means of UV/visible absorption spectra and LS‐55 fluorescence spectrophotometer. NMR spectra were recorded by means of a Bruker Ultrashield 600 Plus NMR spectrometer. ESI‐MS was measured in the Agilent UPLC/Q‐Tof (Agilent 1290 UPLC/6550Q‐TOF). The confocal fluorescence images of the cells were collected using an Olympus FLUOVIEW FV3000 microscope (Olympus Imaging America Inc., Japan), PA computed tomography system equipped with a 10 MHz, 10 mJ cm^−2^, 384‐element ring ultrasound array, and an optical parametric oscillator (OPO) (Surelite II‐20, Continuum, Santa Clara, CA, USA) with 4–6 ns pulse duration and 20 Hz pulse repetition rate used as the light source.

### Synthesis of Type I Photosensitizer BP

Compounds 2 and 4 were prepared according to the documented methods.^1‐2^ Compound 2 (500 mg, 2.51 mmol) was added to the round bottom flask containing methanol (25 mL), compound 4 (300 mg, 1.09 mmol) was added to this reaction mixture, and reflux for 30 min at 80 °C after that silver carbonate (1 g, 3.63 mmol) was added slowly to the reaction mixture and reflux the reaction mixture for 3 h. After that, the reaction mixture was filtered and purified by chromatography, eluting with a gradient (20–50%) of methanol/dichloromethane to give a dark blue solid (100 mg, yield 25.6%). ^1^H NMR (600 MHz, CDCl_3_) δ 9.14 (d, J = 12.0 Hz, 1H), 8.09 (s, 1H), 8.03 (d, J = 12.0 Hz, 1H), 8.01 (d, J = 6.0 Hz, 1H), 7.81 (t, J = 18.0 Hz, 1H), 7.69 (t, J = 18.0 Hz, 2H), 7.24 (d, J = 12.0 Hz, 1H), 3.94 (m, J = 24.0 Hz, 4H), 3.70 (m, J = 18.0 Hz, 4H), 1.50 (t, J = 18.0 Hz, 6H), 1.37 (t, J = 18.0 Hz, 6H). MS (ESI) Calcd for C_24_H_28_N_3_S [M]^+^: 390.20, found: 390.20.

### Synthesis of Hypoxia‐Responsive Type I Photosensitizers BPN 1–5

BP (195 mg, 0.5 mmol) was dissolved in deionized water. Dichloromethane, sodium bicarbonate (84 mg, 1 mmol), and sodium dithionate (263 mg, 0.75 mmol) were added to the solution of BP, stirring for 2 h. When the mixture turned yellow, the dichloromethane containing leuco‐BP was quickly poured into a round bottom flask containing triethylamine (TEA, 85 µL, 0.6 mmol) and a magnetic stirrer. Triphosgene (TPG, 60 mg, 0.16 mmol) in 1 mL dichloromethane was slowly added into the reaction mixtures. Upon completion of dropwise addition, the reaction was stirred at room temperature for 0.5 h. Nitroaryl substrate 1–5 (108 mg, 0.5 mmol) and triethylamine (70 µL, 0.5 mmol) were added to the solution. After an overnight reaction at room temperature, the mixture was collected after washing with water three times. The obtained organic phase was evaporated under reduced pressure. The precipitates were purified by chromatography, eluting with a gradient (20–50%) of ethyl acetate/petroleum ether to obtain target compound BPN 1–5.

BPN 1: A dark solid, 42.8 mg, 15% yield. ^1^H NMR (600 MHz, CDCl_3_) δ 8.25 (d, J = 6.0 Hz, 2H), 7.93 (d, J = 6.0 Hz, 2H), 7.47 (t, J = 18.0 Hz, 3H), 6.81 (s, 1H), 6.69 (s, 2H), 6.62 (d, J = 12.0 Hz, 2H), 5.30 (s, 2H), 3.35 (m, J = 24.0 Hz, 8H), 1.16 (t, J = 12.0 Hz, 12H). MS (ESI) Calcd for C_32_H_34_N_4_O_4_S [M‐H]^−^: 569.22, found: 569.32.

BPN 2: A brown solid, 65.0 mg, 21.6% yield. ^1^H NMR (600 MHz, CDCl_3_) δ 8.42 (d, J = 12.0 Hz, 1H), 8.29 (d, J = 6.0 Hz, 1H), 7.56 (d, J = 12.0 Hz, 1H), 7.52 (d, J = 12.0 Hz, 2H), 7.44 (t, J = 18.0 Hz, 1H), 7.01 (d, J = 12.0 Hz, 1H), 6.94 (d, J = 12.0 Hz, 1H), 6.83 (s, 1H), 6.49 (d, J = 12.0 Hz, 1H), 6.41 (s, 1H), 4.71 (d, J = 72.0 Hz, 2H). 3.47 (s, 3H), 3.29 (m, J = 24.0 Hz, 4H), 3.14 (m, J = 18.0 Hz, 4H), 1.12 (t, J = 12.0 Hz, 6H), 1.03 (t, J = 12.0 Hz, 6H). MS (ESI) Calcd for C_33_H_36_N_4_O_5_S [M+H]^+^: 602.24, found: 602.45.

BPN 3: A brown solid, 53.5 mg, 16.8% yield. ^1^H NMR (600 MHz, CDCl_3_) δ 8.40 (s, 1H), 8.29 (d, J = 6.0 Hz, 1H), 8.23 (d, J = 6.0 Hz, 1H), 8.13 (d, J = 12.0 Hz, 1H), 7.82 (d, J = 12.0 Hz, 1H), 7.49 (t, J = 18.0 Hz, 1H), 7.41 (t, J = 12.0 Hz, 1H), 7.03 (d, J = 12.0 Hz, 1H), 6.96 (s, 1H), 6.50 (s, 1H), 6.46 (d, J = 6.0 Hz, 1H), 5.04 (s, 2H), 3.28 (m, J = 18.0 Hz, 4H), 3.15 (m, J = 24.0 Hz, 4H), 1.11 (t, J = 12.0 Hz, 6H), 1.04 (t, J = 12.0 Hz, 6H). MS (ESI) Calcd for C_33_H_33_F_3_N_4_O_4_S [M+K]^+^: 675.18, found: 675.43.

BPN 4: A blue solid, 25.4 mg, 9.1% yield. ^1^H NMR (600 MHz, CDCl_3_) δ 7.71 (dd, J = 6.0 Hz, 2H), 7.53 (dd, J = 12.0 Hz, 2H), 7.41 (d, J = 6.0 Hz, 3H), 7.34 (d, J = 6.0 Hz, 3H), 5.40 (s, 2H), 4.23 (m, J = 18.0 Hz, 4H), 4.19 (m, J = 18.0 Hz, 4H), 0.92 (t, J = 12.0 Hz, 12H). MS (ESI) Calcd for C_30_H_32_N_4_O_5_S [M]^+^: 560.21, found: 560.45.

BPN 5: A brown solid, 20.1 mg, 5.6% yield. ^1^H NMR (600 MHz, CDCl_3_) δ 8.25 (dd, J = 6.0 Hz, 6H), 7.71 (m, J = 6.0 Hz, 1H), 7.56 (dd, J = 6.0 Hz, 6H), 7.53 (m, J = 12.0 Hz, 1H), 7.35 (d, J = 6.0 Hz, 1H), 7.28 (d, J = 6.0 Hz, 1H), 7.08 (dd, J = 12.0 Hz, 1H), 5.81 (m, J = 36.0 Hz, 2H), 5.35 (m, J = 66.0 Hz, 2H), 5.01 (d, J = 6.0 Hz, 1H), 4.98 (d, J = 6.0 Hz, 1H), 4.94 (d, J = 6.0 Hz, 1H), 4.92 (d, J = 6.0 Hz, 1H), 4.30 (m, J = 24.0 Hz, 1H), 4.23 (m, J = 18.0 Hz, 1H), 4.20 (m, J = 18.0 Hz, 1H), 4.14 (m, J = 18.0 Hz, 1H), 0.88 (t, J = 12.0 Hz, 12H). MS (ESI) Calcd for C_40_H_41_N_5_O_6_S [M+H]^+^: 721.29, found: 721.36.

### General Procedure for NTR Detection

2 mm BPN 2 (10 µL) and 10 mm NADH (100 µL) were mixed in 2 mL PBS. In this way, the obtained BPN 1–5 and NADH concentrations were 10 and 500 µm, respectively. Then the appropriate amount of NTR was added to the above mixed solution. After mixing the solution, it was incubated at 37 °C to monitor the enzyme responsiveness.

The molecular dynamics simulations calculated for the binding affinity between BPN 1–5 and NTR were performed using AMBER20.^[^ ^25^ ^]^ The NTR structure was obtained from the PDB database (PDB ID: 4DN2). The molecular docking results were visualized with PyMOL software.

### Molecular Docking

The protein structure of NTR (PDB ID: 4DN2) was employed and reported as the receptor and executed the molecular docking using Schrodinger 2012. First, the pKa values of ionizable groups in the protein and the protonation/ionization states of the corresponding residues were obtained by the PROPKA program.^[^ ^26^ ^]^ Then, the original ligand in the complex was redocked into the protein with Glide.^[^ ^27^ ^]^ The RMSD between the predicted ligand binding conformation and the original observed binding conformation was ≈0.7 Å, which suggested that Glide was available. As for ligands preparation, the probe molecules were processed by LigPrep panel to produce the corresponding low‐energy 3D structures, which included desalting, hydrogen addition, 3D conformations, coordinates generation, molecular force filed‐based optimization, and ionization states generation at pH 7.3. Next, the processed molecules were docked into the NTR protein with Glide at the SP level. After visual inspection of the docked compounds (with a focus on whether there is hydrogen bond interaction between the nitro group and Arg10‐11 or Ser12), appropriate conformation was selected for following molecular dynamics simulations and free energy calculations.

### System Preparation

Each complex was placed in a water box with TIP3P^[^ ^28^ ^]^ molecules with counter Na^+^ or Cl^+^ to neutralize the system. Restrained electrostatic potential (RESP) charges for each small molecule were obtained by using Gaussian 03 at HF/6‐31G* level and the GAFF force field was used to model the small molecules. The Amber ff14SB force field^[^ ^29^ ^]^ was used for parameterizing the protein.

### Molecular Dynamics Simulation

AMBER20 was used for molecular dynamics simulation. First, a four‐step minimization was performed. Each step of optimization procedures utilized both 2500 steps of the steepest descent method and 2500 steps of the conjugate gradient method, at the same time, position restraints were gradually released. Second, the temperature of each system was increased to 300 K gradually in 50 ps using Langevin dynamics.^[^ ^30^ ^]^ Then, the system underwent a density equilibration with weak restraints on the complex followed by 100 ps constant pressure equilibration with the pressure ≈1 atm maintained by the Parrinello–Rahman pressure coupling algorithm.^[^ ^31^ ^]^ During the above preliminary simulations under both NVT and NPT ensembles, the weight of the restraints was 10 kcal mol^−1^ Å^−2^. Subsequently, the system underwent an 8 ns relaxation under the NPT ensemble to reach a stable conformation, which was then used as the starting point for the subsequent simulations aimed at calculating free energy. All simulations will be run with shake^[^ ^32^ ^]^ on hydrogen atoms, a 2 fs time step, and Langevin dynamics for temperature control, and the particle mesh Ewald (PME) algorithm^[^ ^33^ ^]^ was used to calculate long‐range electrostatic interactions.

### Free Energy Calculation

The last 1 ns trajectory (100 frames) of the previous molecular dynamics simulation was extracted for MM‐PBSA calculations. The internal energy of the system in a vacuum was calculated using the Amber ff14SB force field. The electrostatic solvation‐free energy was calculated with the semi‐empirical radius parameters optimized by Tan and Luo,^[^ ^34^ ^]^ and the nonpolar solvation‐free energy (*G*_np_) contribution was calculated by the following equation, in which the ΔSASA is the solvent‐accessible surface area.

(1) $$G_{np}=\;0.00542\times \Delta SASA+0.9200$$

### Selectivity Assay

2 mm BPN 2 (10 µL), 10 mm NADH (100 µL) in 2 mL PBS, and an appropriate volume of interfering substances were mixed: PBS, NTR(10 µg mL^−1^), Cys (1 mm), GSH (10 mm), NaHS (1 mm), H_2_O_2_ (1 mm), vitamin C (Vc, 1 mm), NaClO (10 µm), NaCl (50 mm), KCl (50 mm), CaCl_2_ (50 mm), glucose (10 mm), Na_2_S_2_O_3_ (1 mm). After 1 h, the solution was measured with corresponding fluorescence (Em = 720 nm).

### Evaluation of PDT Efficacy In Vitro

For superoxide radical detection, the solution of BPN 2 treated with/without NTR and DHR123 (10 µm) was prepared. Then the above solutions were exposed to 680 nm pulse‐wave irradiation (0.5 W cm^−2^) at different times, and the fluorescence spectra were detected (Em: 525 nm). DHR123 aqueous solution as control was measured. For hydroxyl radical detection, the solution of BPN 2 treated with/without NTR and benzoic acid (BA) (10 µm) was prepared. Then the above solutions were exposed to 680 nm pulse‐wave irradiation (0.5 W cm^−2^) at different times, and the fluorescence spectra were recorded (Em: 420 nm). BA aqueous solution as the negative control and H_2_O_2_ (50 µm) + different concentrations of Fe^2+^ as the positive control were measured. In addition, DMPO was applied as the spin trap agent for OH•. ESR spectroscopy was employed to detect the ESR signals of the above groups of samples. For singlet oxygen detection, the solution of BPN 2 treated with/without NTR and SOSG (5 µm) was prepared. Then the above solutions were exposed to 680 nm pulse‐wave irradiation (0.5 W cm^−2^) at different times, and the fluorescence spectra were detected (Em: 525 nm). SOSG aqueous solution as control was measured.

### Cytotoxicity Testing

MTT assay was carried out to evaluate the toxicity. Experimental Mammary Tumor‐6 (EMT6) cells were seeded in a 96‐well plate, which was incubated at 37 °C for 24 h. First, the cytotoxicity of CoCl_2_ was investigated. EMT6 cells were incubated with different concentrations of CoCl_2_ for 6 h, and then the cell viability was measured. Next, the cytotoxicity of BPN 2 was investigated. EMT6 cells were incubated with varying concentrations of BPN 2 (0, 5, 10, 15, 20, and 25 µm) for another 24 h, and then the cell viability was measured. The phototoxicity of BPN 2 was measured. The hypoxia EMT6 cells were incubated with varying concentrations of BPN 2 for 4 h. The old media were removed and cells were rinsed twice by PBS. Then, the irradiation group was exposed under 680 nm pulse‐wave irradiation (0.5 W cm^−2^, 5 min), and the cell viability was detected. The cell viability was determined via the equation (*n* = 5).

### Confocal Fluorescence Imaging

EMT6 cells were seeded in 35 mm confocal culture dishes, which were incubated at 37 °C for 24 h. Then, the EMT6 cells were divided randomly into five groups. Two groups were treated with or without BPN 2 (25 µm) for 4 h. Other groups were treated with different concentrations of CoCl_2_ for 6 h and were further incubated with BPN 2. The cells were imaged by a confocal laser scanning microscope (CLSM) (Ex: 640 nm, Em: 670–700 nm).

### Intracellular O_2_^−•^ Imaging

EMT6 cells were seeded in 35 mm confocal culture dishes, which were incubated at 37 °C for 24 h. Then, the EMT6 cells were divided randomly into six groups. Two groups were treated with or without BPN 2 (25 µm) for 4 h. Other groups were treated with different concentrations of CoCl_2_ for 6 h and were further incubated BPN 2. The above cells were further cultured by DHE (10 µm, 30 min). After all treatments, the cells were exposed to 680 nm pulse‐wave irradiation (0.5 W cm^−2^, 5 min). The red fluorescence of DHE was imaged using CLSM (Ex: 488 nm, Em: 570–630 nm).

### PDT Efficiency of the BPN 2 on EMT6 Cells

EMT6 cells were seeded in 35 mm confocal culture dishes, which were incubated at 37 °C for 24 h. Then, EMT6 was exposed to different following treatments: pretreated with PBS (control), Laser, BPN 2, BPN 2 + PW, 50 µm CoCl_2_ + BPN 2 + PW, 50 µm CoCl_2_ + BPN 2 + CW (continuous‐wave). Afterward, the cells were stained with calcein AM/PI and were imaged by CLSM.

### Photocavitation Damage of BPN 2 in Cells

EMT6 cells were seeded in 35 mm confocal culture dishes, which were incubated at 37 °C for 24 h. Then, EMT6 were treated with 50 µm CoCl_2_ for 6 h and were further exposed to different following treatments. The green fluorescence of DCFH and the cellular morphology were imaged using CLSM (Ex: 488 nm, Em: 500–550 nm).

### Flow Cytometry Analysis

The EMT6 cells were seeded in the six‐well plates, which were incubated at 37 °C for 24 h. After different treatments, the cells were stained and determined by flow cytometry (Becton Dickinson, Mountain View, CA, USA).

### Tumor Mouse Model

All animal procedures were performed in accordance with the National Institutes of Health (NIH) Guidelines for the Care and Use of Laboratory Animals of South China Normal University, and the experiments were approved by the Animal Ethics Committee of South China Normal University. 4‐week‐old male BALB/c mice were purchased from the Animal Experiment Center, Southern Medical University, and bred in an axenic environment. Mouse was injected with 1 × 10^6^ of EMT6 cells to develop tumors with a volume of ≈100 mm^3^ (Tumor volume = the greatest longitudinal diameter (length) × the greatest transverse diameter (width)^2^ × 0.5).

### Blood Biochemistry and Hematology Analysis

The healthy mice (*n* = 3) were intravenously injected with BP (PBS, 100 µL, 1 mm) and BPN 2 (PBS‐DMSO solution (99%, v/v), 100 µL, 1 mm). Then the blood samples were collected at 7 days post injection and then sent for blood analysis.

### In Vivo Imaging

EMT6 tumor‐bearing mice were intravenously injected with BPN 2 (PBS‐DMSO solution (99%, v/v), 100 µL, 1 mm) or BP (100 µL, 1 mm). For FL imaging, a NIR imaging system (Odyssey LI‐COR, USA) was used for observing the mice at different intervals (continuous laser, Ex: 680 nm, Em: 720 nm). For PA imaging, a PA computed tomography system was used for observing the mice at different intervals (pulsed laser, Ex: 680 nm).

### Imaging of Tumor Tissue after Injection of BPN 2

EMT6 tumor‐bearing mice were sacrificed after injection of BPN 2 for 12 h to obtain the tumor tissues. After sectioning with a cryostat, CLSM was used to observe the fluorescence signal of BPN 2 in the tissue. (Ex = 640 nm, Em = 655–755 nm).

### Evaluation of Phototherapeutic Efficacy In Vivo

EMT6 tumor‐bearing mice were randomly separated into six groups (*n* = 5). The control groups were treated with PBS. The Laser groups were treated with the 680 nm PW irradiation (0.5 W cm^−2^, 10 min). The BPN 2 groups were treated with BPN 2 (PBS‐DMSO solution (99%, v/v), 100 µL, 1 mm) via intravenous injection. The BPN 2 + CW groups were treated with BPN 2 (PBS‐DMSO solution (99%, v/v), 100 µL, 1 mm) for 12 h along with the 680 nm CW irradiation (0.5 W cm^−2^, 10 min). The BPN 2 + Vc + PW groups were treated with Vc (50 µL, 500 µm) by intratumoral and BPN 2 (PBS‐DMSO solution (99%, v/v), 100 µL, 1 mm) for 12 h along with the 680 nm PW irradiation (0.5 W cm^−2^, 10 min), the test groups were treated with BPN 2 (PBS‐DMSO solution (99%, v/v), 100 µL, 1 mm) for 12 h along with the 680 nm PW irradiation (0.5 W cm^−2^, 10 min). The body weights and tumor sizes of mice were then evaluated.

### Histology Examination of the Tumor

The tumor tissues and major organs (including the heart, liver, spleen, lung, and kidney) were peeled off from the sacrificed mice after 18 days of treatment. The tissues were fixed with 4% paraformaldehyde and then sliced into sections. All the harvested tissues were used for H&E staining and TUNEL staining.

### Statistical Analysis

Data were expressed as mean ± standard deviation. Two‐tailed Student's *t*‐test was used to evaluate the statistical significance. *p* values < 0.05 were regarded statistically significant (*p < 0.05, **p < 0.01, ***p < 0.001).

## Conflict of Interest

The authors declare no conflict of interest.

## Supporting information

Supporting Information

## Data Availability

The data that support the findings of this study are available from the corresponding author upon reasonable request.
