# Supplementary material for: Totally Caged Type I Pro‐Photosensitizer for Oxygen‐Independent Synergistic Phototherapy of Hypoxic Tumors
Source: Adv Sci (Weinh). 2024 Jun 17;11(31):2400462. doi: 10.1002/advs.202400462 (PMC11336908; doi:10.1002/advs.202400462)
Supplement: Supplementary file 1 — Supporting Information [file ADVS-11-2400462-s001.docx]

Supporting Information

Totally Caged Type I Pro-Photosensitizer for Oxygen-Independent Synergistic Phototherapy of Hypoxic Tumors

Qin Zeng, Xipeng Li, Jiajun Li, Mengting Shi, Yufen Yao, Lei Guo,* Na Zhi, and Tao Zhang*

Qin Zeng, Xipeng Li, Jiajun Li, Mengting Shi, Na Zhi, Tao Zhang

MOE Key Laboratory of Laser Life Science & Institute of Laser Life Science, Guangdong Provincial Key Laboratory of Laser Life Science, College of Biophotonics, South China Normal University Guangzhou 510631, People’s Republic of China

Qin Zeng

The Seventh Affiliated Hospital Southern Medical University Foshan, Guangdong 528244, China

Yufen Yao, Lei Guo

School of Pharmaceutical Sciences, Sun Yat-sen University, Guangzhou 510006, China

Tao Zhang

Guangzhou Key Laboratory of Spectral Analysis and Functional Probes, College of Biophotonics, South China Normal University, Guangzhou 510631, China

^*^Email: guolei7@mail.sysu.edu.cn (L. Guo); zt@scnu.edu.cn (T. Zhang)


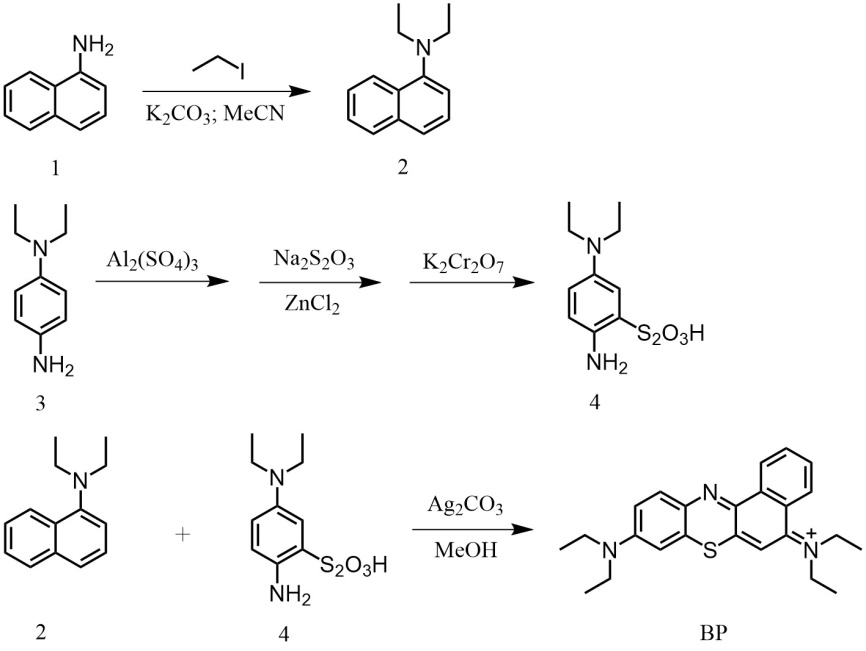


Figure S1 Synthetic route of the compound BP.


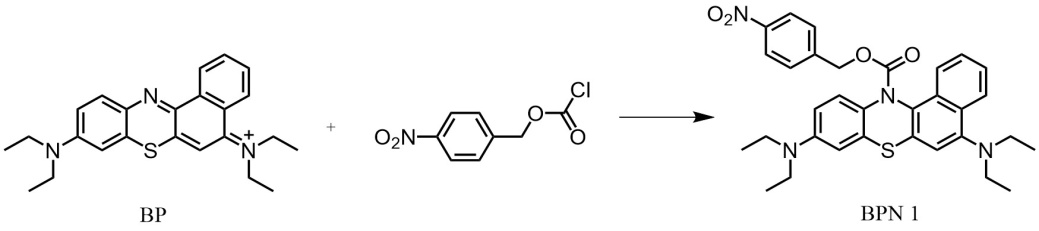


Figure S2 Synthetic route of the compound BPN 1.


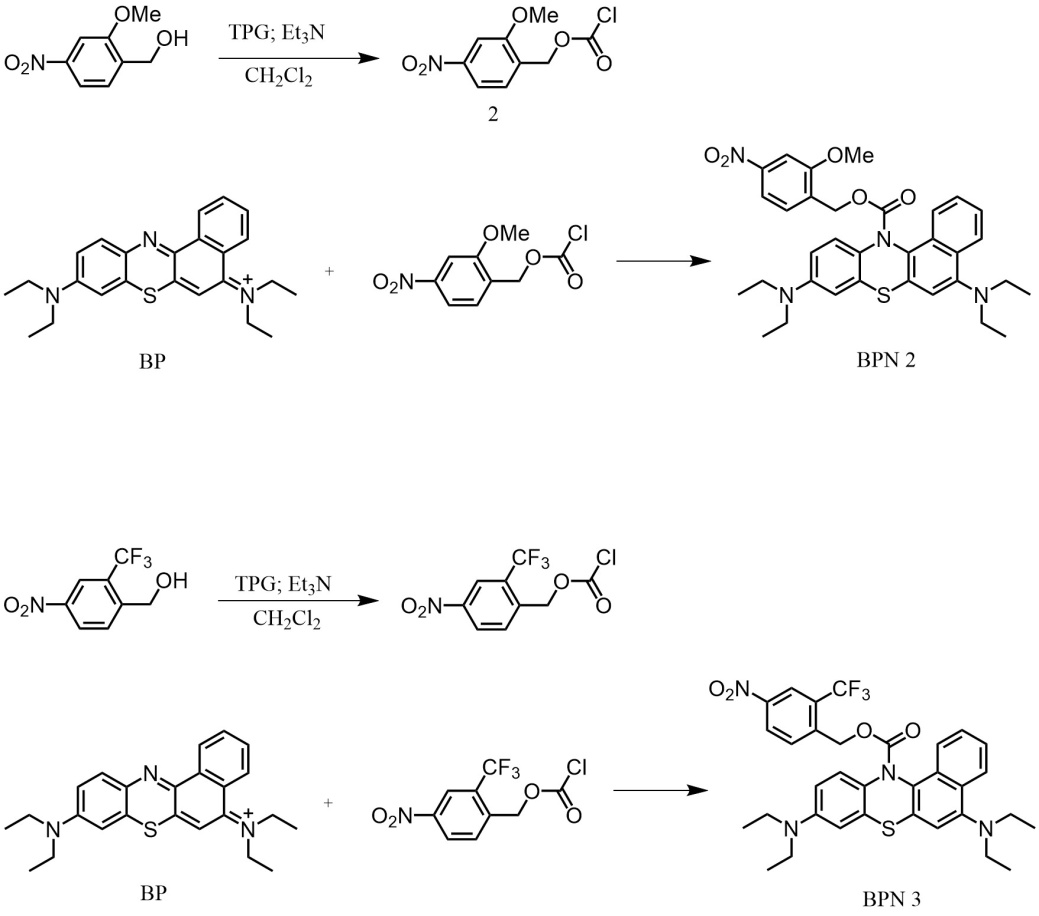


Figure S3 Synthetic route of the compound BPN 2.


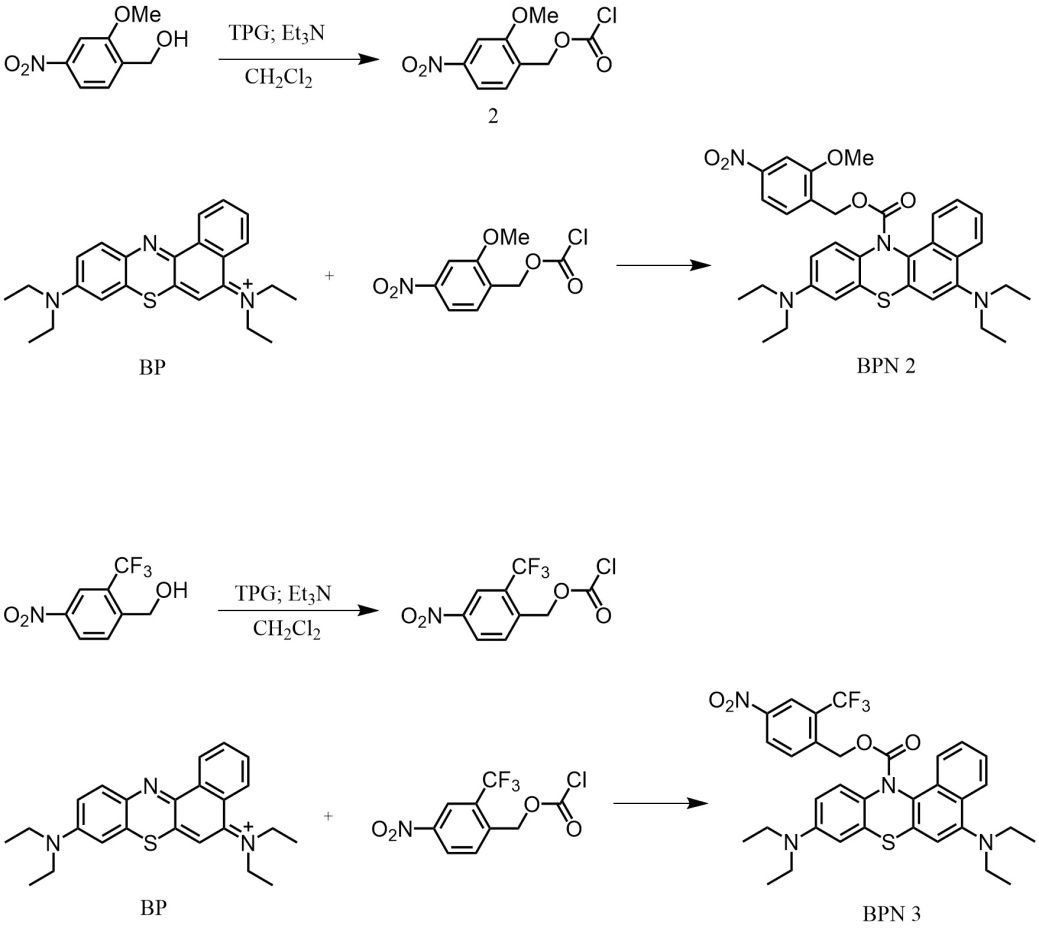


Figure S4 Synthetic route of the compound BPN 3.


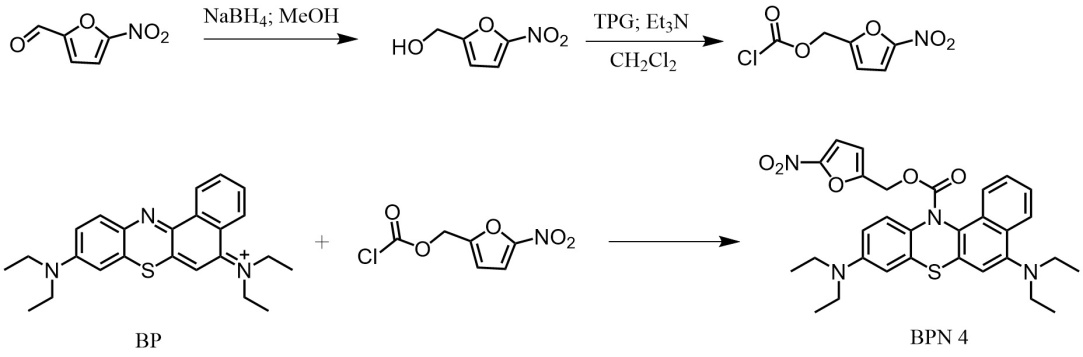


Figure S5 Synthetic route of the compound BPN 4.


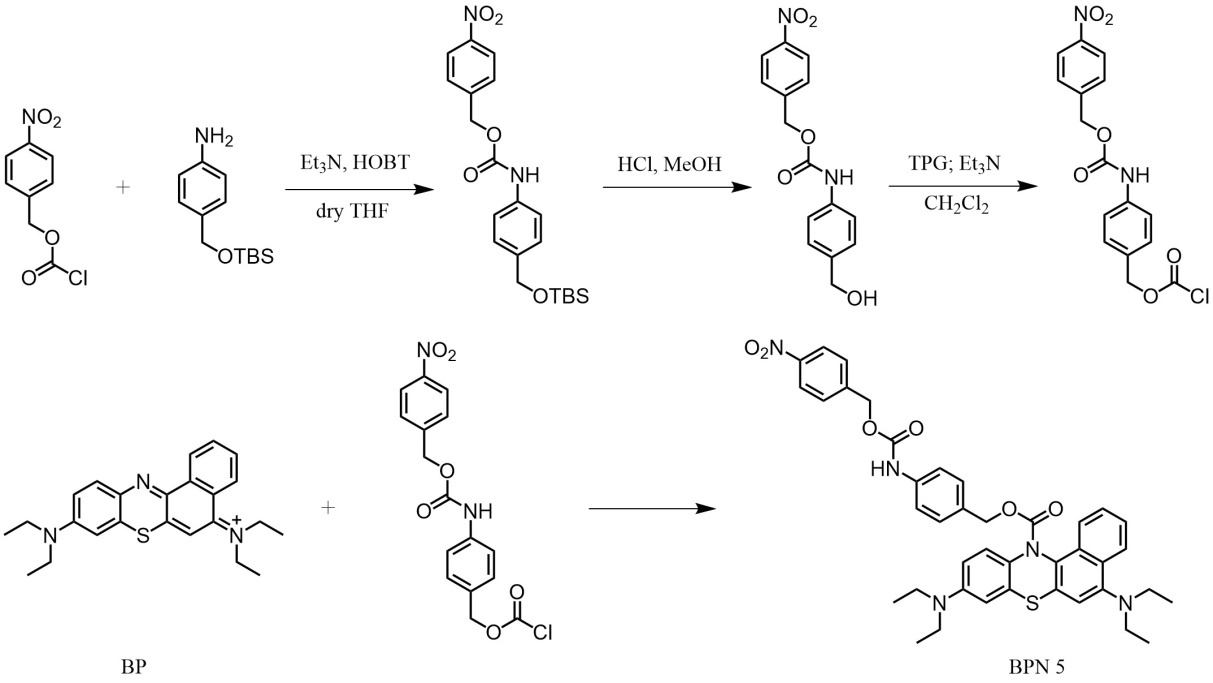


Figure S6 Synthetic route of the compound BPN 5.


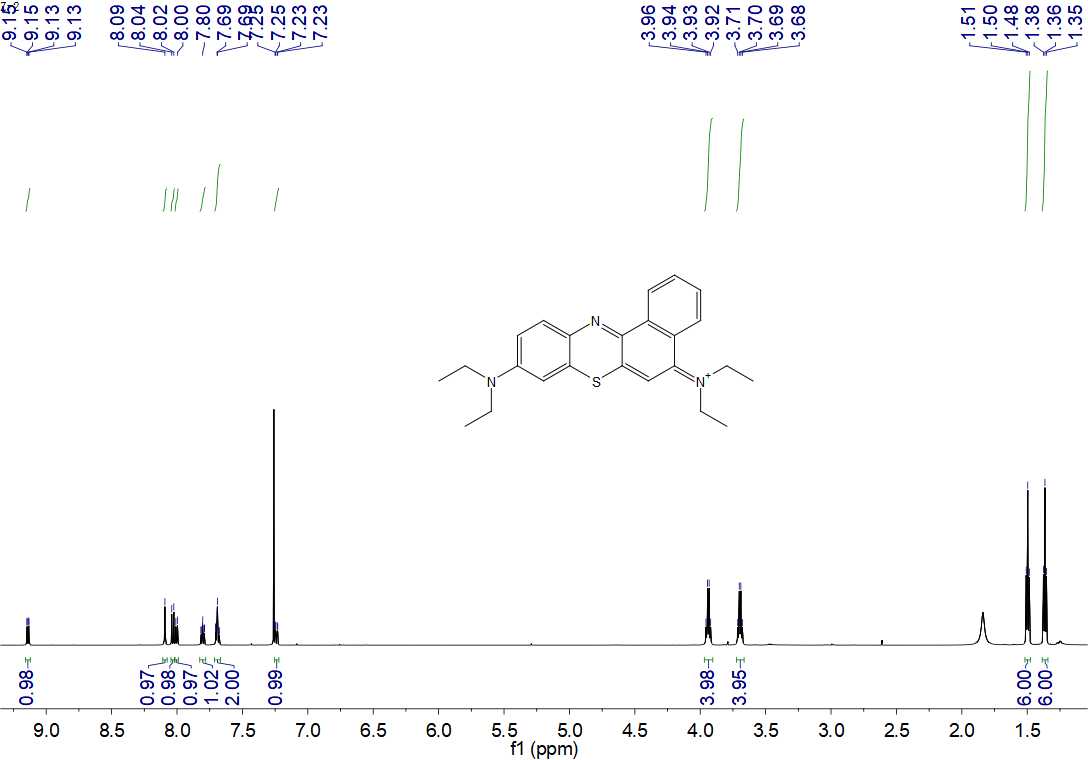


Figure S7 ^1^H NMR spectra of compound BP in CDCl_3_.


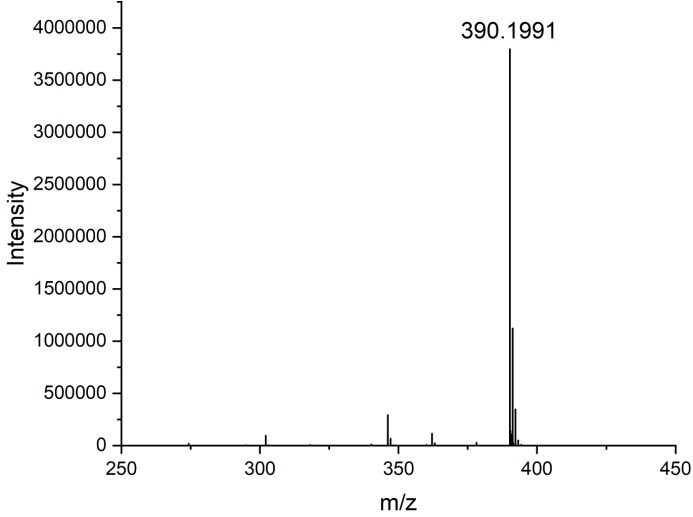


Figure S8 ESI-MS spectrum of compound BP.


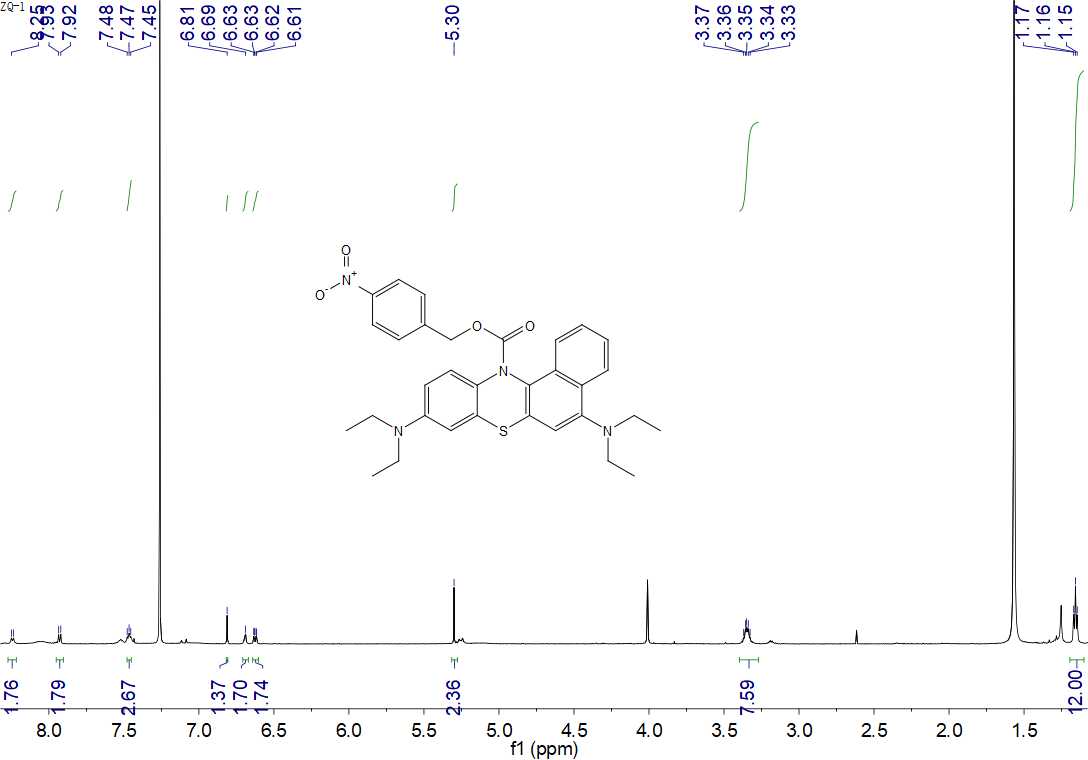


Figure S9 ^1^H NMR spectra of compound BPN 1 in CDCl_3_.


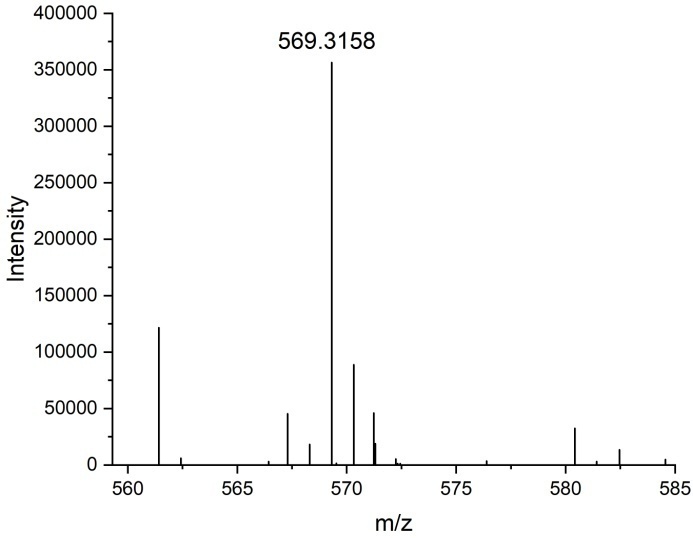


Figure S10 ESI-MS spectrum of compound BPN 1.


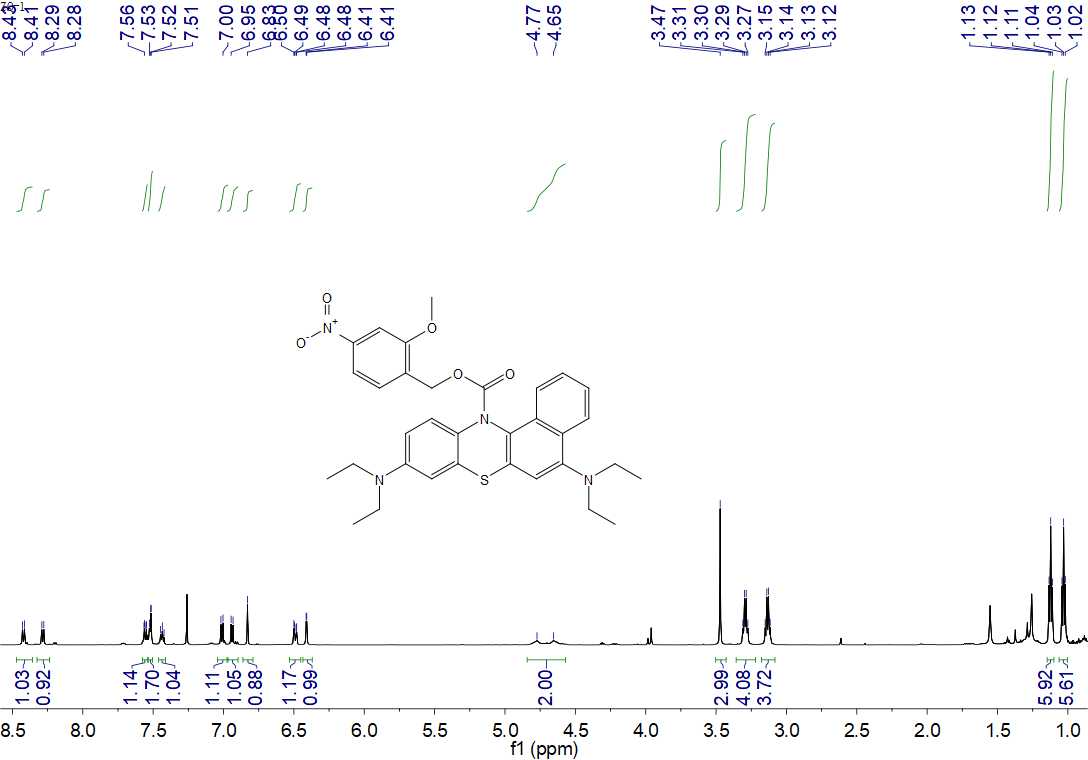


Figure S11 ^1^H NMR spectra of compound BPN 2 in CDCl_3_.


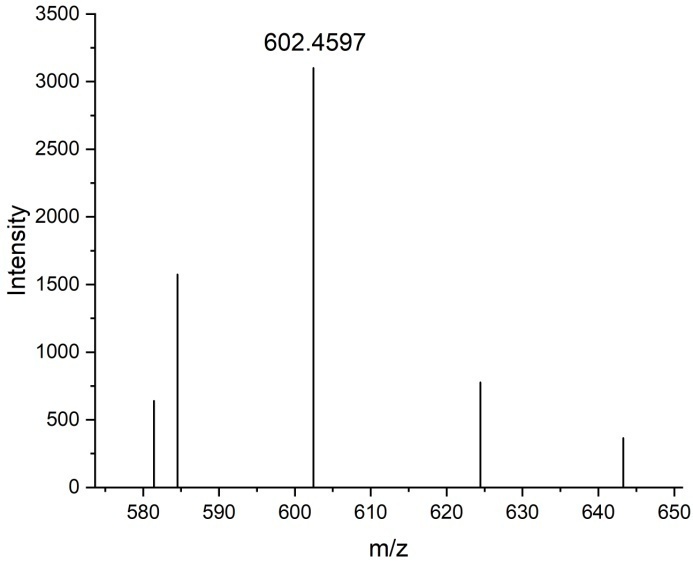


Figure S12 ESI-MS spectrum of compound BPN 2.


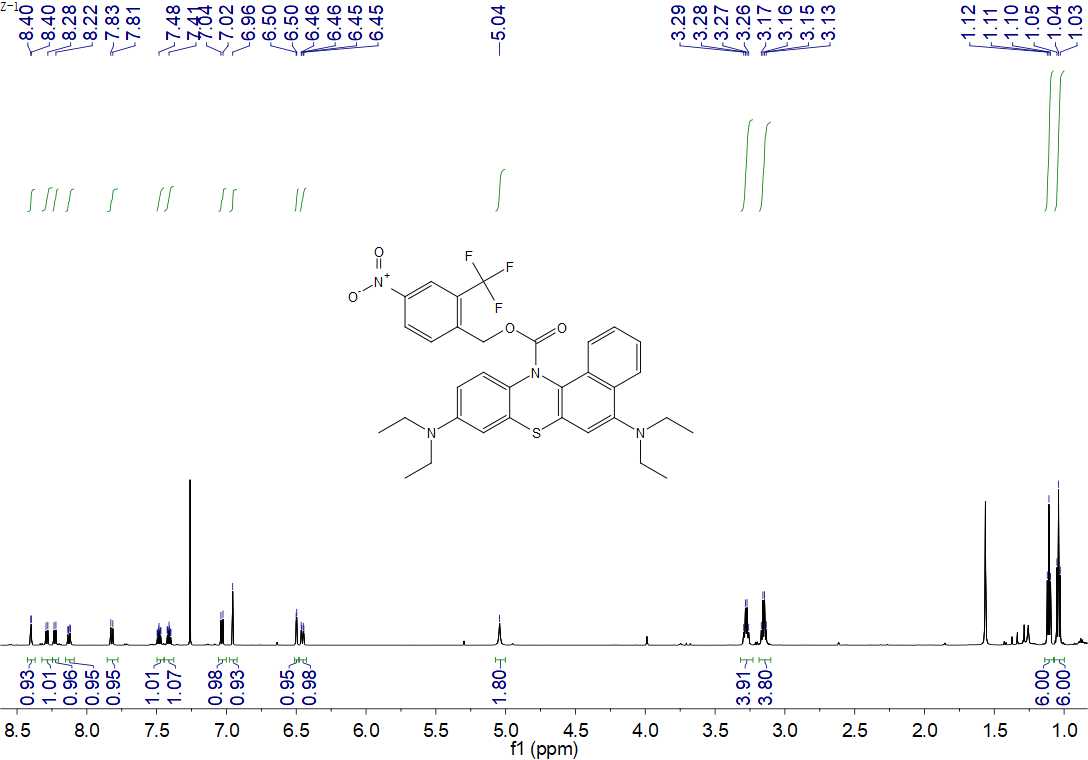


Figure S13 ^1^H NMR spectra of compound BPN 3 in CDCl_3_.


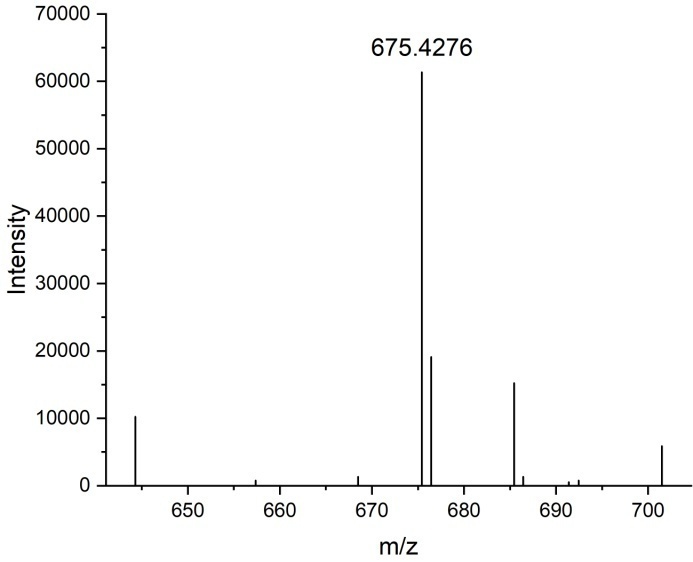


Figure S14 ESI-MS spectrum of compound BPN 3.


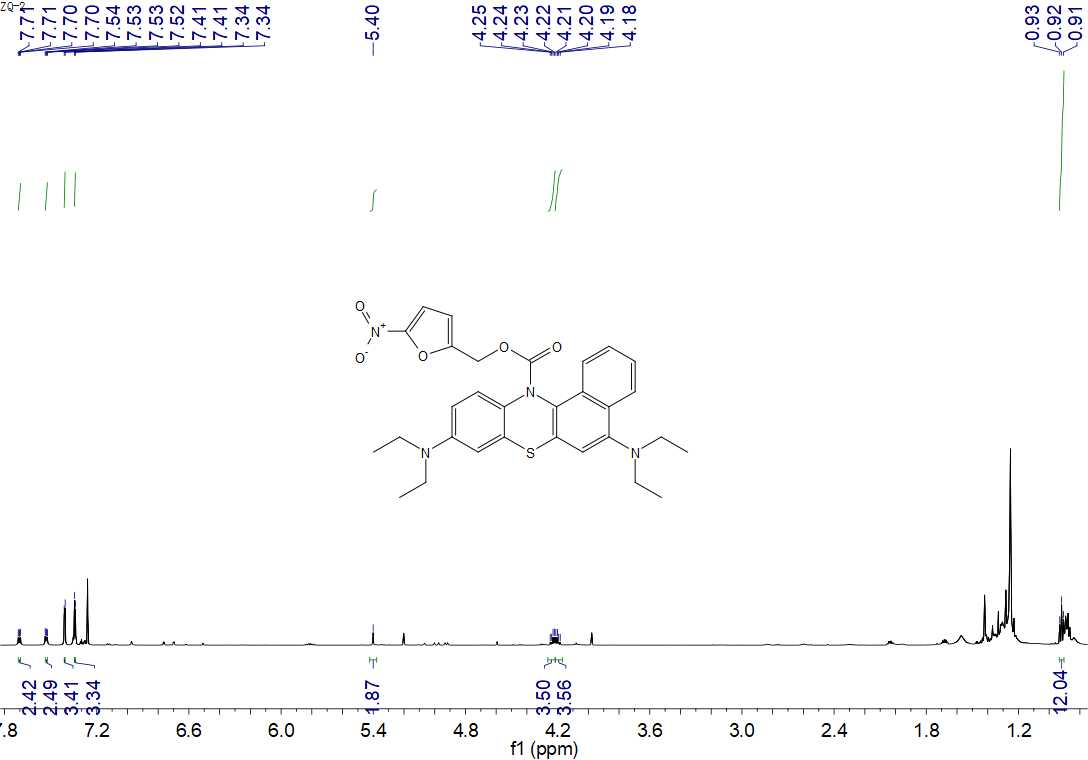


Figure S15 ^1^H NMR spectra of compound BPN 4 in CDCl_3_.


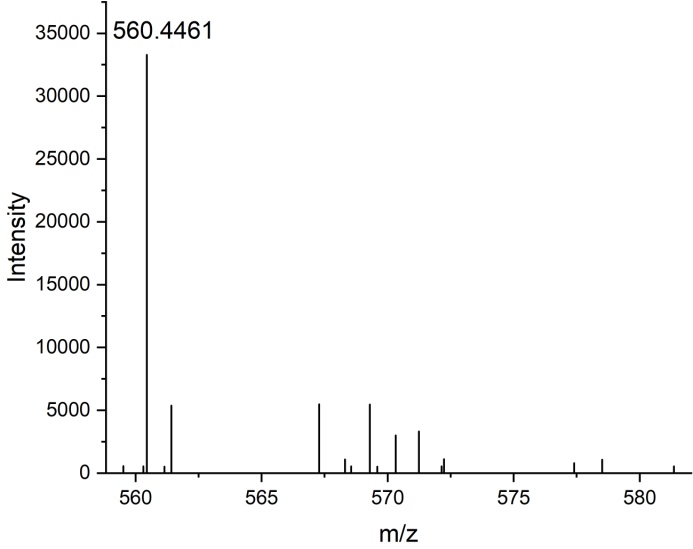


Figure S16 ESI-MS spectrum of compound BPN 4.


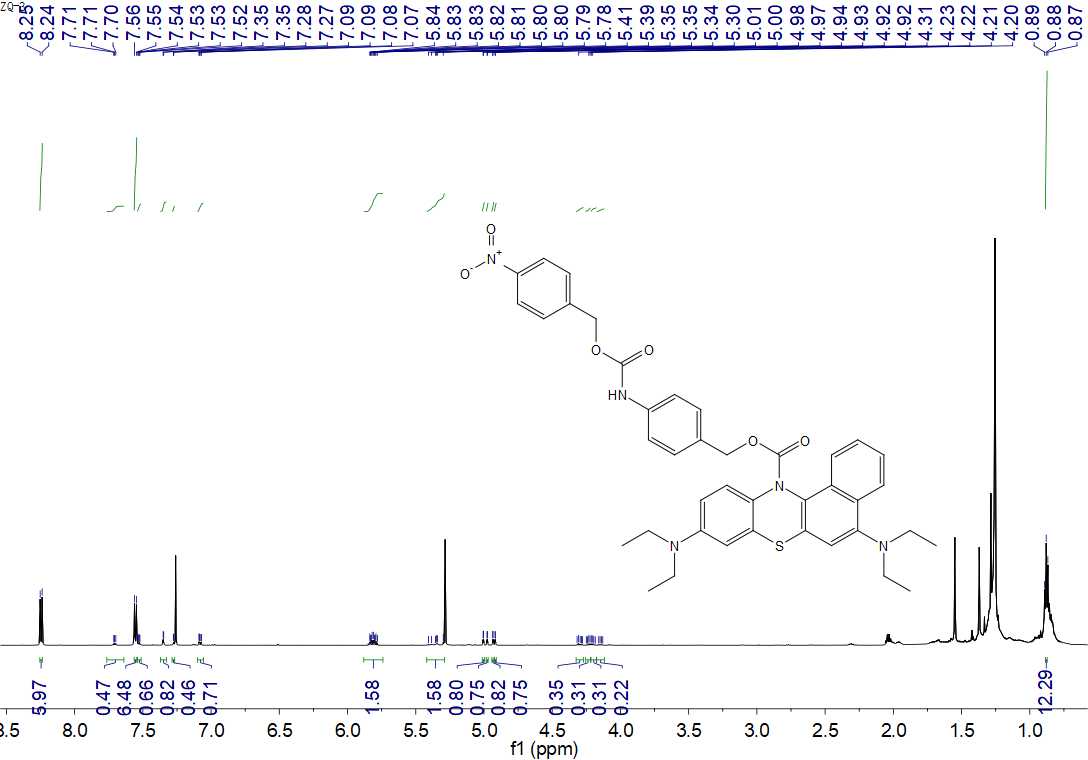


Figure S17 ^1^H NMR spectra of compound BPN 5 in CDCl_3_.


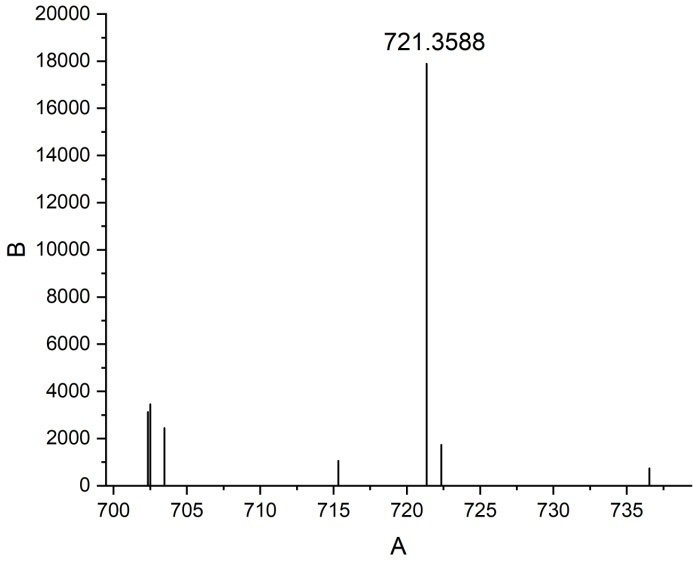


Figure S18 ESI-MS spectrum of compound BPN 5.


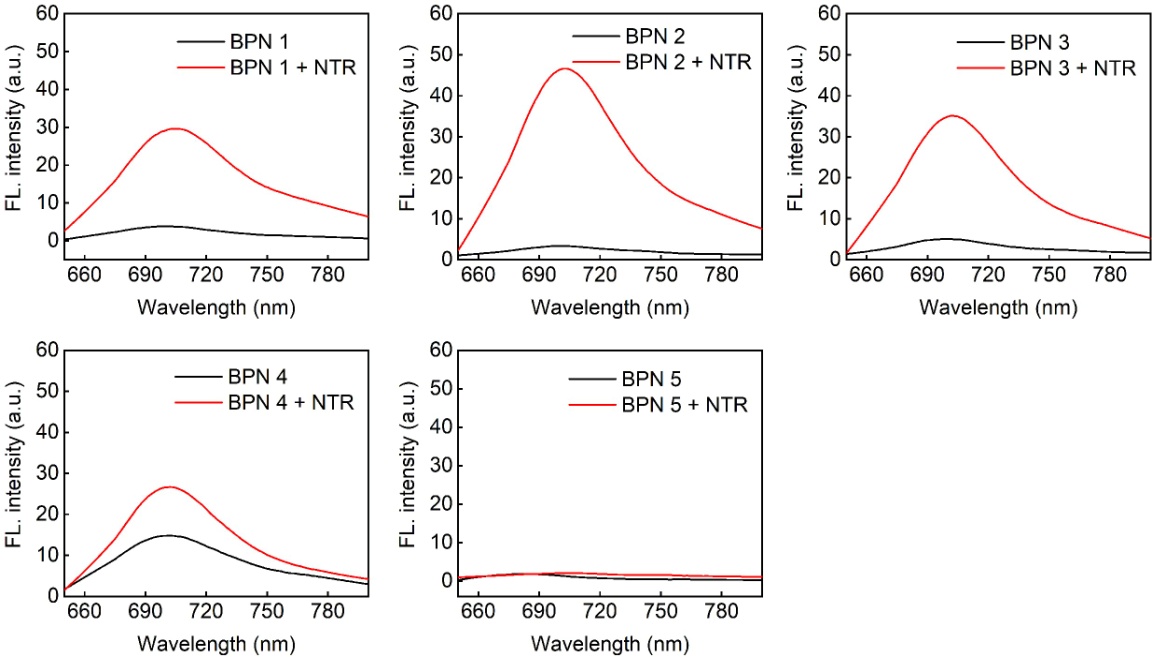


Figure S19 Fluorescence spectra of BPN 1-5 (10 μM) before and after reaction with NTR (10 μg/mL).


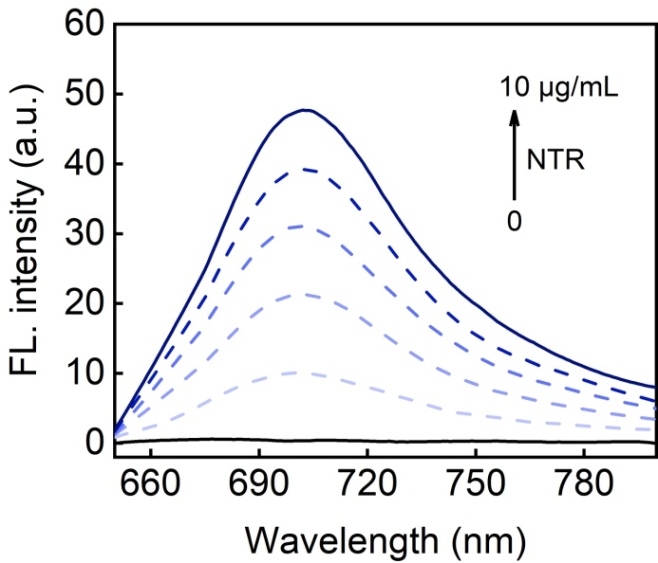


Figure S20 Fluorescence spectra of BPN 2 (10 μM) towards varied concentrations of NTR.


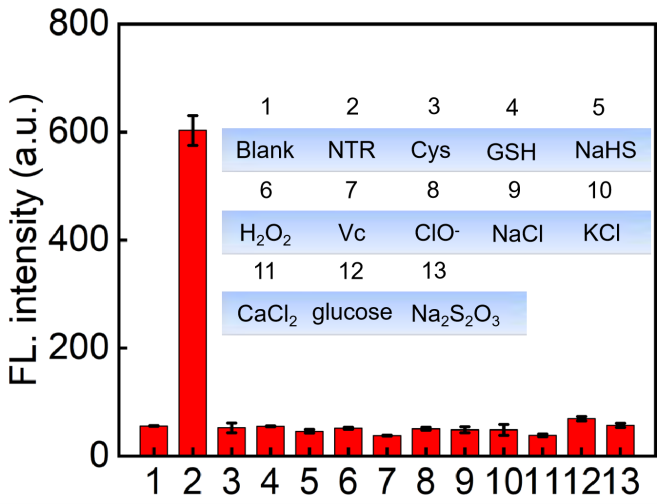


Figure S21 Fluorescence responses of BPN 2 (10 μM) toward different kinds of species: Blank (BPN 2 + NADH), NTR (10 μg/mL), Cys (1 mM), GSH (10 mM), NaHS (1 mM), H_2_O_2_ (1 mM), Vitamin C (1 mM), NaClO (10 μM), NaCl (50 mM), CaCl_2_ (50 mM), KCl (50 mM), glucose (10 mM), Na_2_S_2_O_3_ (1 mM).


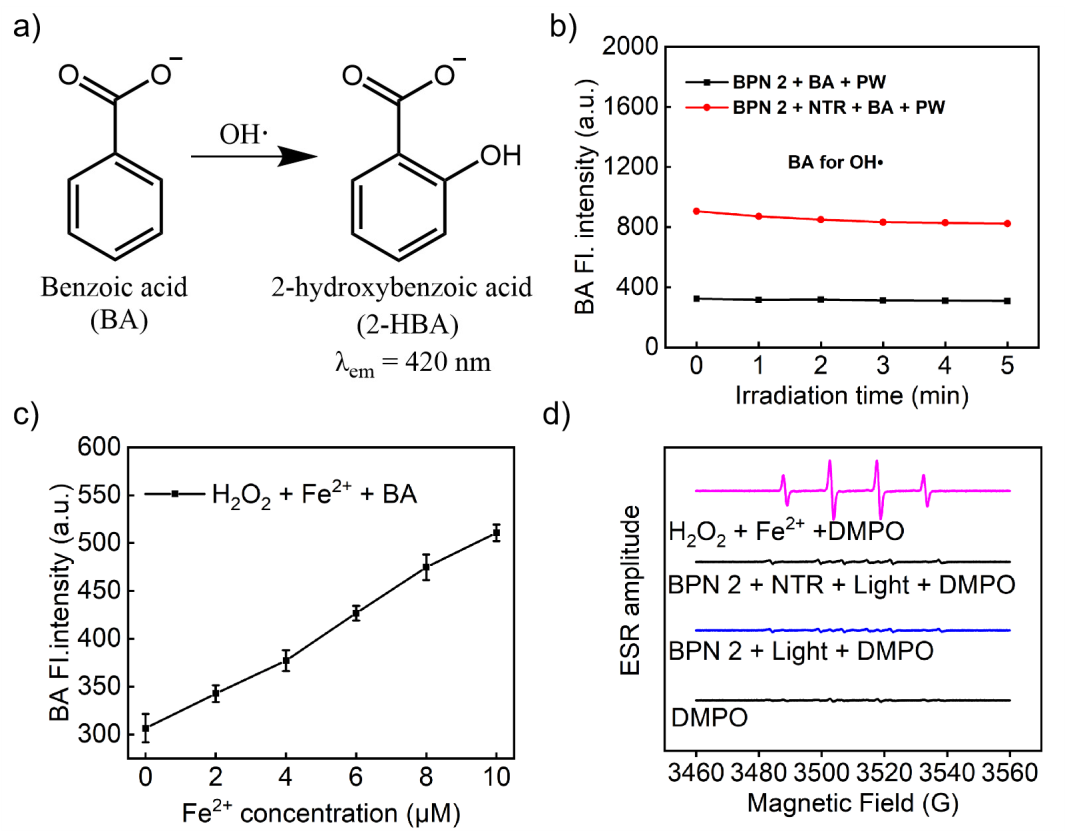


Figure S22 a) The mechanism of BA as the OH• scavenger monitors OH• generation in the solution. Fluorescence spectra of BA (10 μM) at b) BPN 2 with or without NTR under light irradiation and c) H_2_O_2_ (50 μM) + different concentrations of Fe^2+^. d) ESR spectra to detect OH• generated, using 5, 5 dimethyl-1-pyrroline-N-oxide (DMPO) as spin-trap agent.


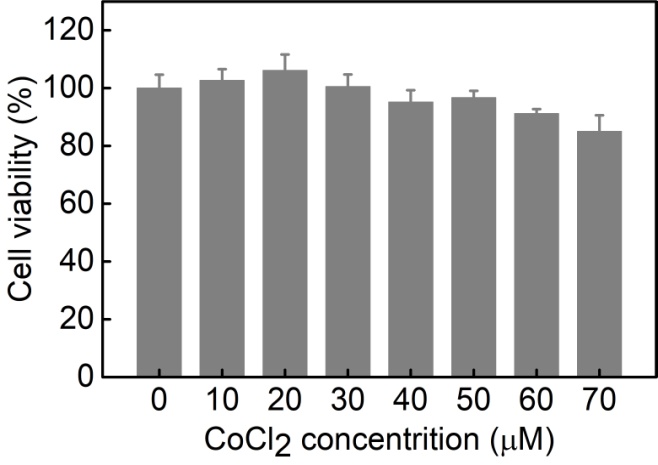


Figure S23 The cell viabilities of EMT6 cells after incubation with various concentrations of cobalt chloride.


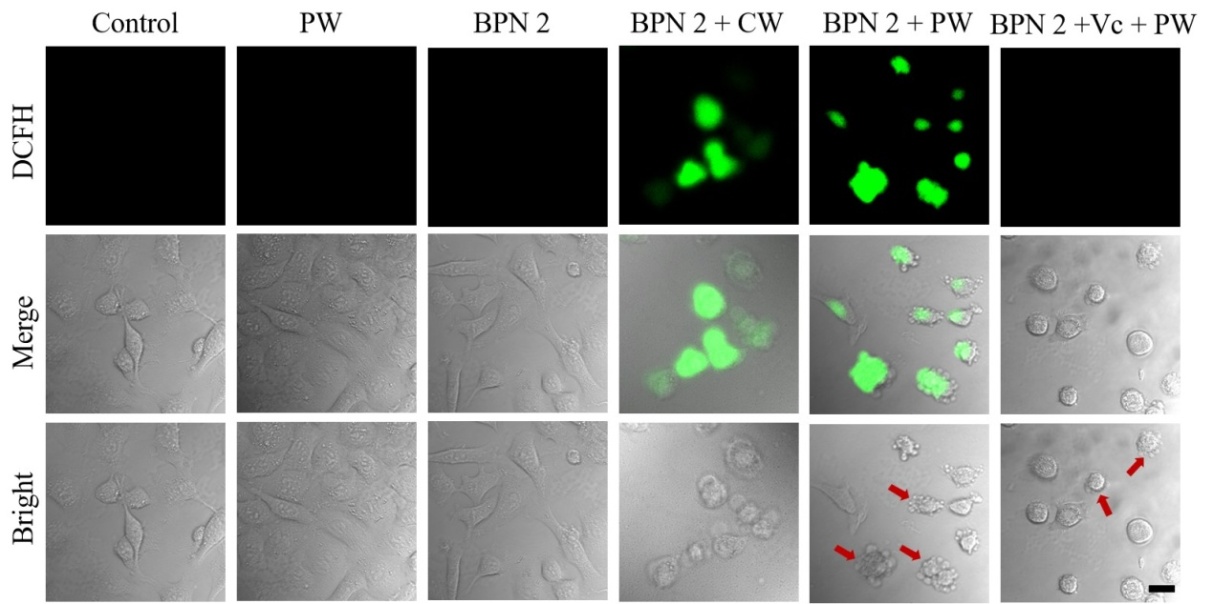


Figure S24 Confocal imaging showing the explosion effect of PW-excited cavitation and the ROS generation on cells. Scale bar = 20 μm.


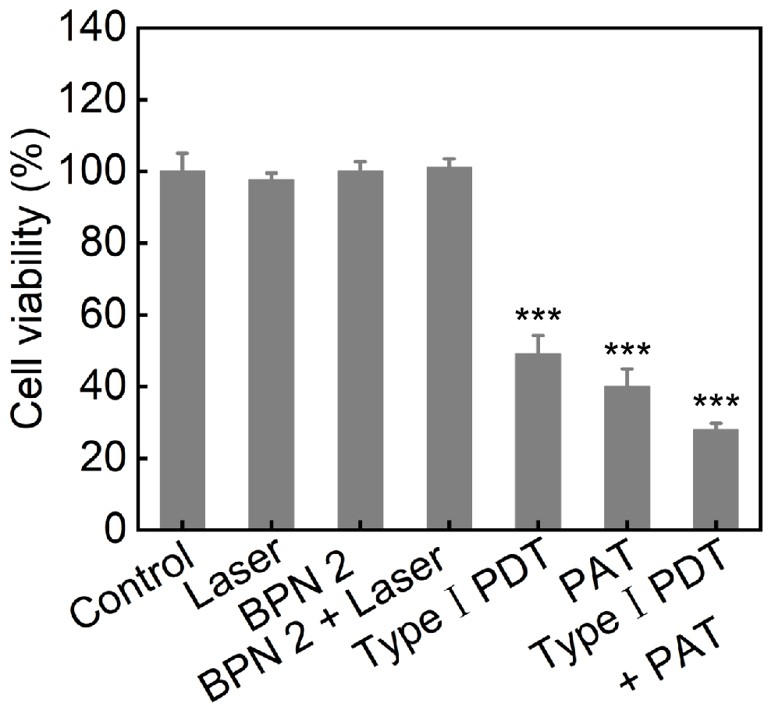


Figure S25 The cell viabilities of EMT6 cells with different treatments to distinguish the role of Type Ⅰ PDT, PAT and Type Ⅰ PDT + PAT.


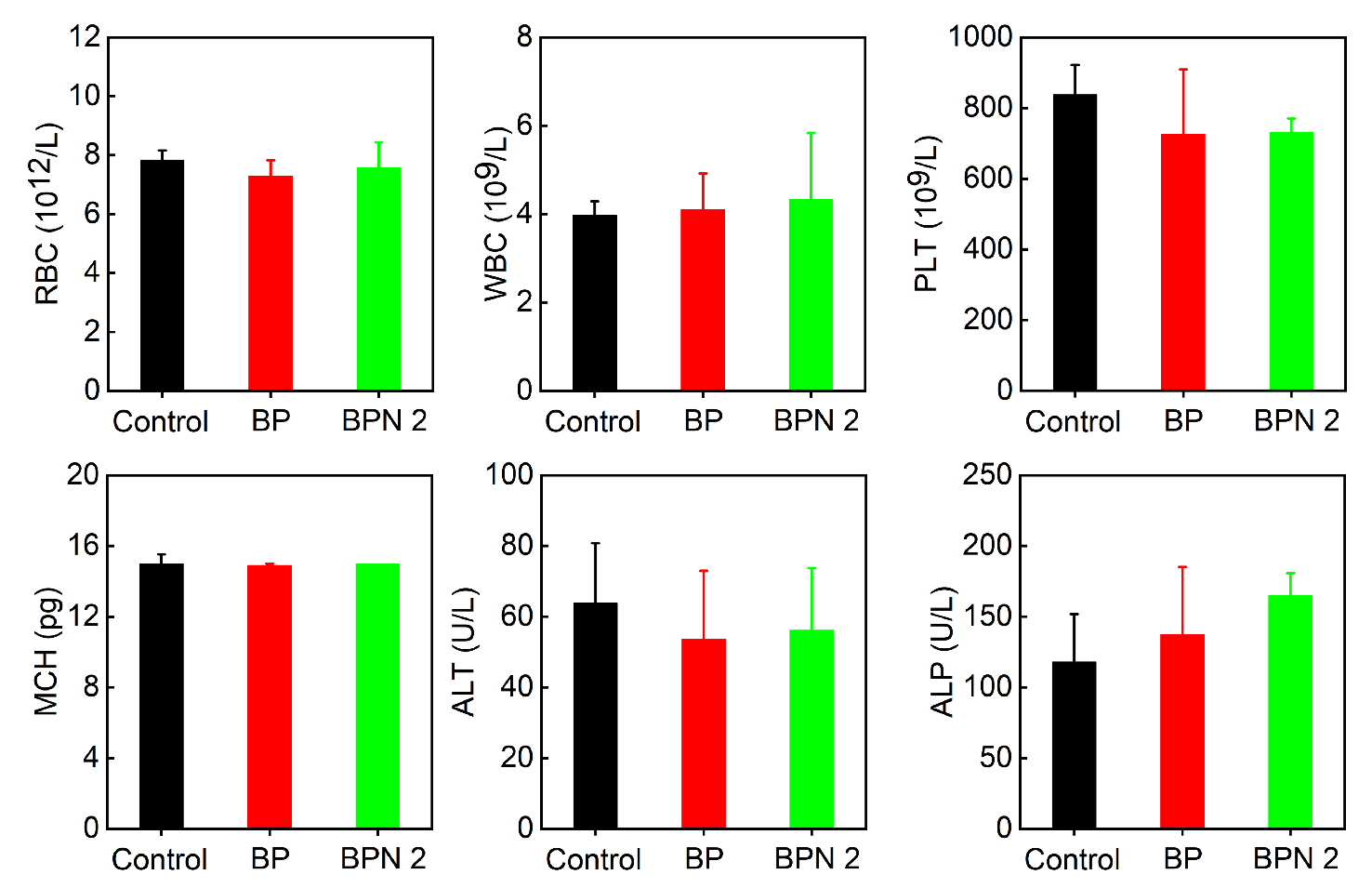


Figure S26 Blood biochemistry and hematology analyses were performed before (0 day) and after intravenous injection with BP and BPN 2 for 7 days, respectively (n=3). The serum biochemical and hematological parameters including red blood count (RBC), white blood count (WBC), platelets (PLT), mean corpuscular hemoglobin (MCH), alanine transaminase (ALT), and alkaline phosphatase (ALP).


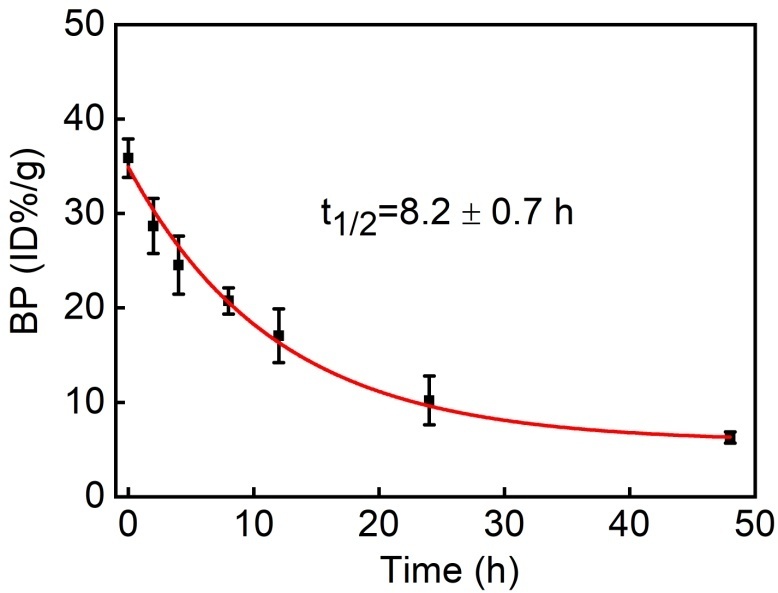


Figure S27 The blood circulation curve of BPN 2 in mice by measuring the absorbance of BP in blood at different time points post i.v. injection (n=5).


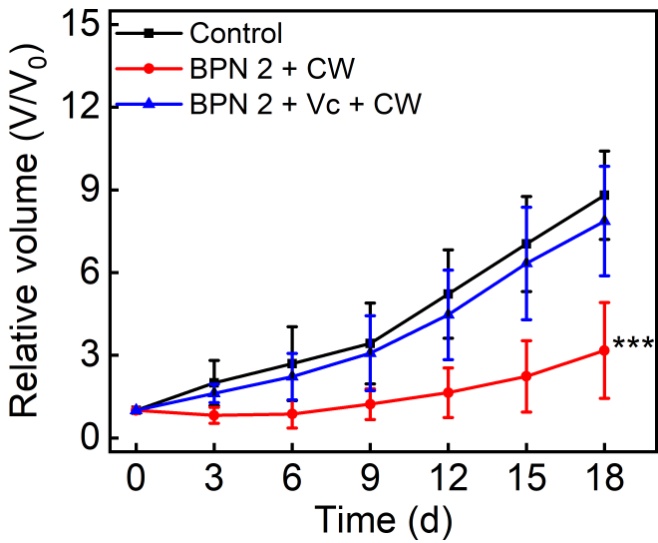


Figure S28 Change of relative tumor volume (V/V_0_) after mice were intravenously injected with PBS, BPN 2 or BPN 2 + Vc and irradiated with 680 nm CW laser at 0.5 W/cm^2^ for 10 min.


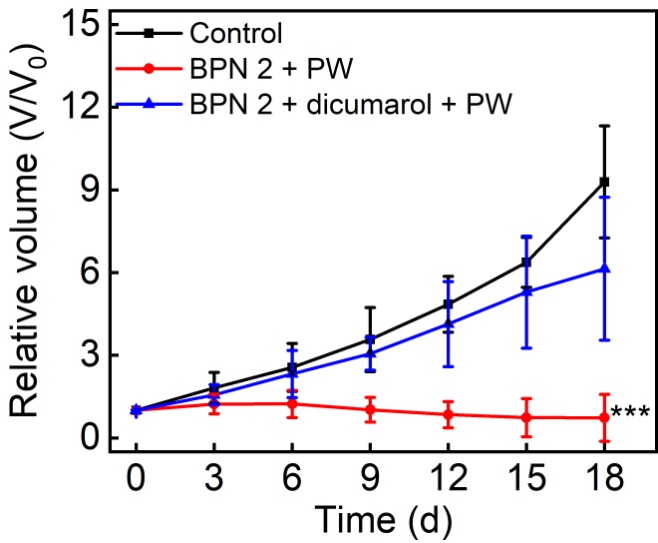


Figure S29 Change of relative tumor volume (V/V_0_) after mice were intravenously injected with PBS, BPN 2 or BPN 2 + dicumarol and irradiated with 680 nm PW laser at 0.5 W/cm^2^ for 10 min.


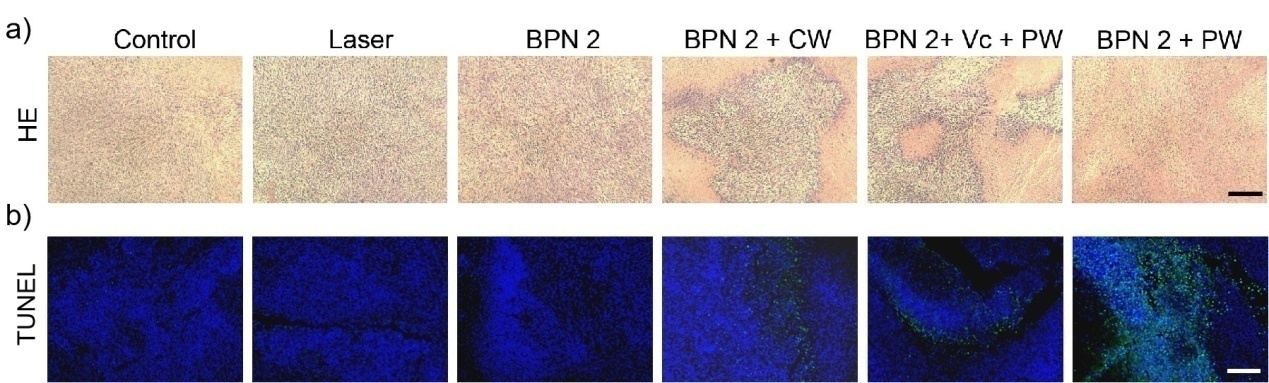


Figure S30 a) H&E and b) TUNEL staining of tumor tissues harvested from the corresponding mice on the 18 day after treatment. Scale bars = 100 μm.


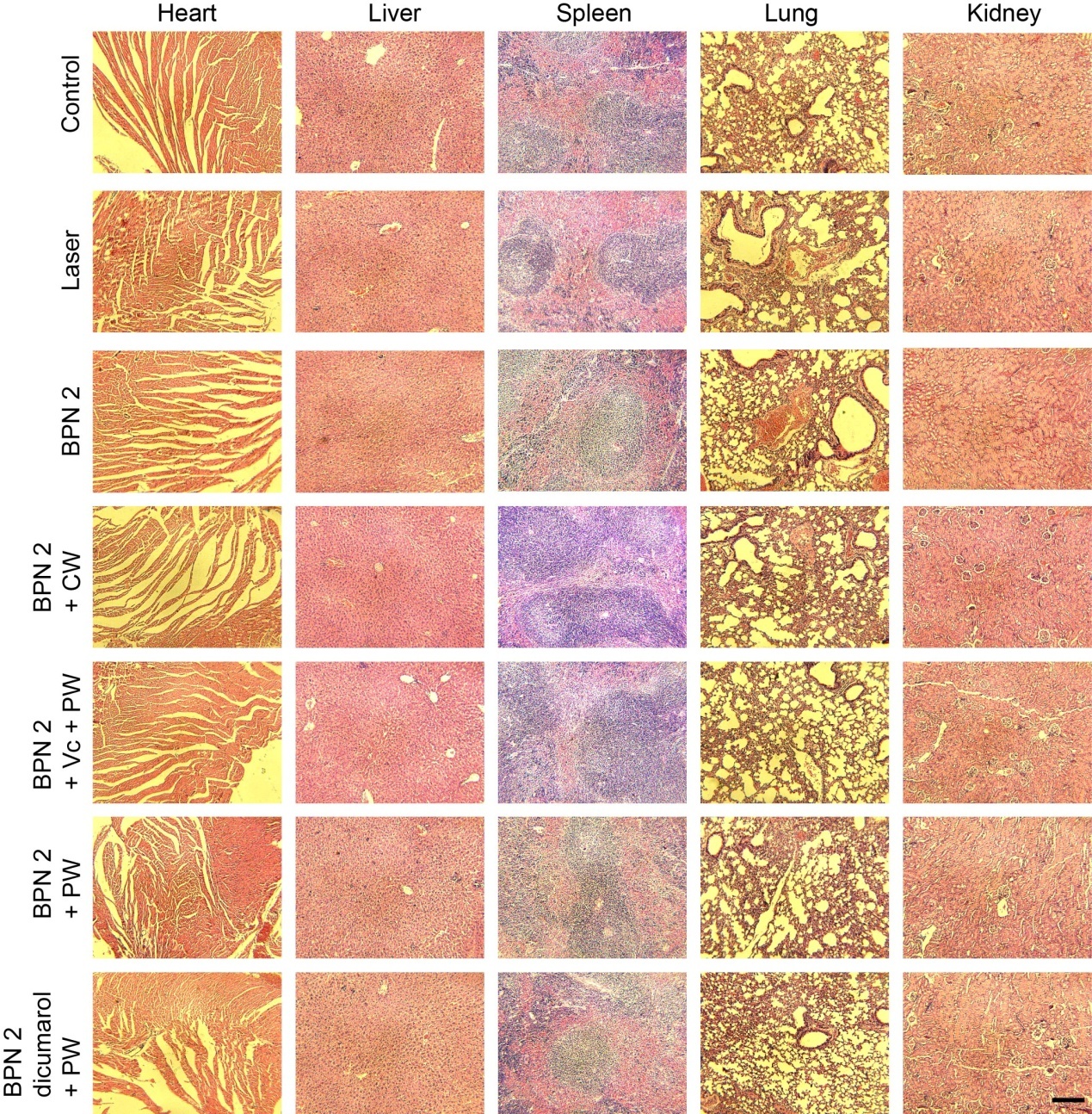


Figure S31 H&E staining of the main body organs (heart, liver, spleen, lung, kidneys) harvested from the corresponding mice on the 18 day after treatment. Scale bars = 100 μm.
